# Heterologous Tissue Culture Expression Signature Predicts Human Breast Cancer Prognosis

**DOI:** 10.1371/journal.pone.0000145

**Published:** 2007-01-03

**Authors:** Eun Sung Park, Ju-Seog Lee, Hyun Goo Woo, Fenghuang Zhan, Joanna H. Shih, John D. Shaughnessy, J. Frederic Mushinski

**Affiliations:** 1 Laboratory of Genetics, Center for Cancer Research, National Cancer Institute, National Institutes of Health, Bethesda, Maryland, United States of America; 2 Laboratory of Experimental Carcinogenesis, Center for Cancer Research, National Cancer Institute, National Institutes of Health, Bethesda, Maryland, United States of America; 3 Biometric Research Branch, Division of Cancer Treatment and Diagnosis, National Cancer Institute, National Institutes of Health, Bethesda, Maryland, United States of America; 4 Donna and Donald Lambert Laboratory of Myeloma Genetics, Myeloma Institute for Research and Therapy, University of Arkansas for Medical Sciences, Little Rock, Arkansas, United States of America; South African National Bioinformatics Institute, South Africa

## Abstract

**Background:**

Cancer patients have highly variable clinical outcomes owing to many factors, among which are genes that determine the likelihood of invasion and metastasis. This predisposition can be reflected in the gene expression pattern of the primary tumor, which may predict outcomes and guide the choice of treatment better than other clinical predictors.

**Methodology/Principal Findings:**

We developed an mRNA expression-based model that can predict prognosis/outcomes of human breast cancer patients regardless of microarray platform and patient group. Our model was developed using genes differentially expressed in mouse plasma cell tumors growing *in vivo* versus those growing *in vitro*. The prediction system was validated using published data from three cohorts of patients for whom microarray and clinical data had been compiled. The model stratified patients into four independent survival groups (BEST, GOOD, BAD, and WORST: log-rank test p = 1.7×10^−8^).

**Conclusions:**

Our model significantly improved the survival prediction over other expression-based models and permitted recognition of patients with different prognoses within the estrogen receptor-positive group and within a single pathological tumor class. Basing our predictor on a dataset that originated in a different species and a different cell type may have rendered it less sensitive to proliferation differences and endowed it with wide applicability.

**Significance:**

Prognosis prediction for patients with breast cancer is currently based on histopathological typing and estrogen receptor positivity. Yet both assays define groups that are heterogeneous in survival. Gene expression profiling allows subdivision of these groups and recognition of patients whose tumors are very unlikely to be lethal and those with much grimmer outlooks, which can augment the predictive power of conventional tumor analysis and aid the clinician in choosing relaxed vs. aggressive therapy.

## Introduction

Cancers are complex tissues whose behavior is strongly influenced by dynamic interactions between the cancer cells, the tumor's stromal cells and the extracellular matrix [1]. Stromal cells provide growth factors, blood supply, and mechanical support, and changes in this microenvironment can trigger tissue remodeling, setting the stage for tumor progression, invasion and metastasis. Since invasion and metastasis require tumor cells to survive and grow in sites quite different from the milieu in which they arose, we reasoned that adaptation of tumor cells to growth *in vitro* might require analogous changes in cell physiology, probably mirrored by changes in gene expression. Thus, we undertook the comparison of gene expression profiles between mouse plasma cell tumors (PCTs) growing in mice and PCTs that had been adapted to growth in tissue culture, hoping to gain insights into the genes responsible for the adaptation of this particular tumor to tissue culture conditions. Another goal for this study, which provides the basis for the present paper, was to determine whether these data might be extrapolatable to other tumor types and other species. More particularly, we hypothesized that the alterations in gene expression required for tumor cells to survive *in vitro* might be markers of human cancers that were particularly suited to growth in distant sites, i.e., more likely to invade or metastasize, two processes associated with poor prognosis and foreshortened survival. Specifically, we sought to test whether expression data from an experimental cancer model in mice, in this case plasma cell tumors, has the potential of uncovering survival/prognosis patterns in human cancers by transcending species-specific and cell lineage-specific gene expression patterns.

Cancer patients have highly variable clinical outcomes based on many factors including the genetic make-up of the patient, the genetic and phenotypic variability of the tumors and the way the tumors interact with their surrounding stroma. It is likely that this spectrum of clinical courses may also reflect different tumor-specific genetic predispositions to metastasize and gene expression heterogeneity that are incompletely recognized by classical diagnosis methods such as histopathological tumor typing and staging. This genetic predisposition might be reflected in specific patterns of gene expression, and it has long been hoped that microarray profiling of tumors' global gene expression could help identify subgroups of patients that differ in prognosis or in their response to available therapeutic modalities [2]–[9]. The ultimate goal is that gene expression profiles of a new patient's tumor could be analyzed in the context of a database of gene expression profiles from patients with known outcomes. In this way, treatment could be more precisely tailored to this patient's expected prognosis and predicted response to treatment.

We generated a mouse plasma cell tissue culture (PCT-TC) gene signature by comparing and contrasting the global gene expression of solid mouse plasma cell tumors with that of plasma cell tumors adapted to grow in tissue culture. We then used these signatures in meta-analysis of published reports of human breast cancer patients that included extensive long-term follow–up and survival data along with microarray data from these cancers. We devised three prediction models by which our PCT-TC signature identified subgroups of patients that could be stratified by their different survivals. In this way we identified and validated the existence of four distinct prognostic groups of breast cancer patients with significant differences in clinical outcomes. This method is superior to previously published expression-based survival prediction and may eventually be useful in predicting prognosis of new patients presenting with this disease.

## Results

### Generation of mouse tissue culture signature

**Figure 1:**
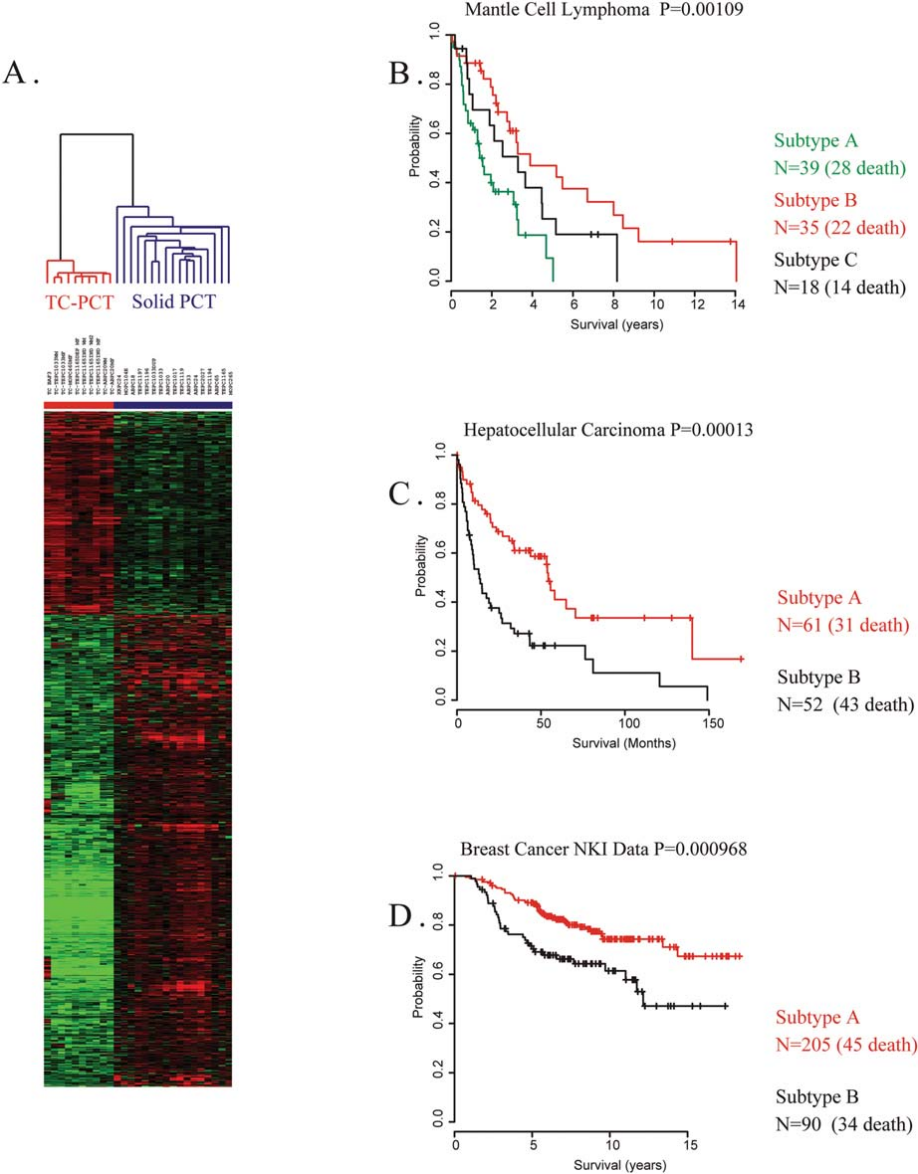
Mouse plasma cell tumor tissue culture (PCT-TC) signature and survival analysis of human cancer patients 27 RNA samples from 17 solid mouse plasma cell tumors and 10 tissue cultured mouse PCT-TC cell lines (including Baf3, a pre-B cell line) were used for the generation of PCT-TC signature. **A.** Mouse plasma cell tumor tissue culture signature. 1162 genes showing significant differences in expression between solid PCTs and tissue-cultured PCTs were selected by SAM analysis at the 99-percentile confidence level with a 0.001 FDR. **B–D.** Kaplan-Meier survival analysis of human cancer patients groups generated by unsupervised cluster analysis with mouse PCT-TC signature. **B.** Survival analysis of human mantle cell lymphoma patients group [12] generated by unsupervised cluster analysis with 694 orthologs of the mouse PCT-TC signature genes. **C.** Survival analysis of human liver cancer patients [13] generated by unsupervised cluster analysis with 971 orthologs of the mouse PCT-TC signature genes. **D.** Survival analysis of human breast cancer patients [2], [3], [15] generated by unsupervised cluster analysis with 470 orthologs of the mouse PCT-TC signature genes.

For the generation of the PCT-TC signature, microarray-based global gene expression analysis was performed on 27 individual RNA samples composed of 17 solid mouse PCTs and 10 tissue cultured PCT cell lines using Affymetrix U74Av2 microarray chips. We applied Significance Analysis of Microarrays (SAM) at the 99 percentile confidence level, and 1162 genes with a 0.001 False Discovery Rate (FDR) emerged as a signature that characterized the differences in gene expression between these two groups. Cluster analysis of these SAM-filtered genes revealed that most solid tumors showed similar expression patterns and clustered together, while the tissue cultured tumor cells clustered together, separated from their *in vivo* growing solid tumor counterparts regardless of tumor induction protocols (Fig. 1A). Approximately 70% of these genes showed lower expression in the cells grown *in vitro* (indicated in green on the heat map in Fig. 1A) compared with the solid tumors. Most of the genes that showed significantly lower expression in cells growing *in vitro* encode genes involved in angiogenesis, chemotaxis, component of extracellular matrix, complement activation, or cell motility-related genes, while the genes higher in expression in tissue cultured conditions are genes related to cell survival (see Table S1). Since these gene families had been cited in reports analyzing tumor invasion and metastasis [10], [11], tumor-progression processes associated with poor prognosis and reduced survival; we decided to test this expression signature on the analysis of human cancer survival based on global gene expression patterns.

### Cross-comparison of gene expression data between mouse plasma cell tumors with three sets of human cancer gene expression data

We initially applied our PCT-TC signature to three human datasets in an attempt to stratify tumor patients into homogeneous groups that might reflect prognosis. Using only orthologous1Homologous murine and human genes that have diverged from each other as a consequence of speciation. Cf., http://www.informatics.jax.org/userdocs/homology_criteria.shtml genes that showed significant difference of expression in SAM analysis of mouse PCT *in vivo* and *in vitro*, unsupervised hierarchical cluster analysis was performed with gene expression data from patients with mantle cell lymphoma [12], hepato-cellular carcinoma [13], and breast cancer [2], independently. Kaplan-Meier plots and log-rank test for the patients clustering together based on similarities in gene expression, revealed significantly different survival estimates in each of these three data sets (Fig. 1B–D). Thus, the molecular differences that reflect tissue culture adaptation appeared to be associated with distinct differences in the clinical outcomes of patients with diverse human cancers.

### Construction and validation of prediction models for human breast cancer patient prognosis

**Figure 2:**
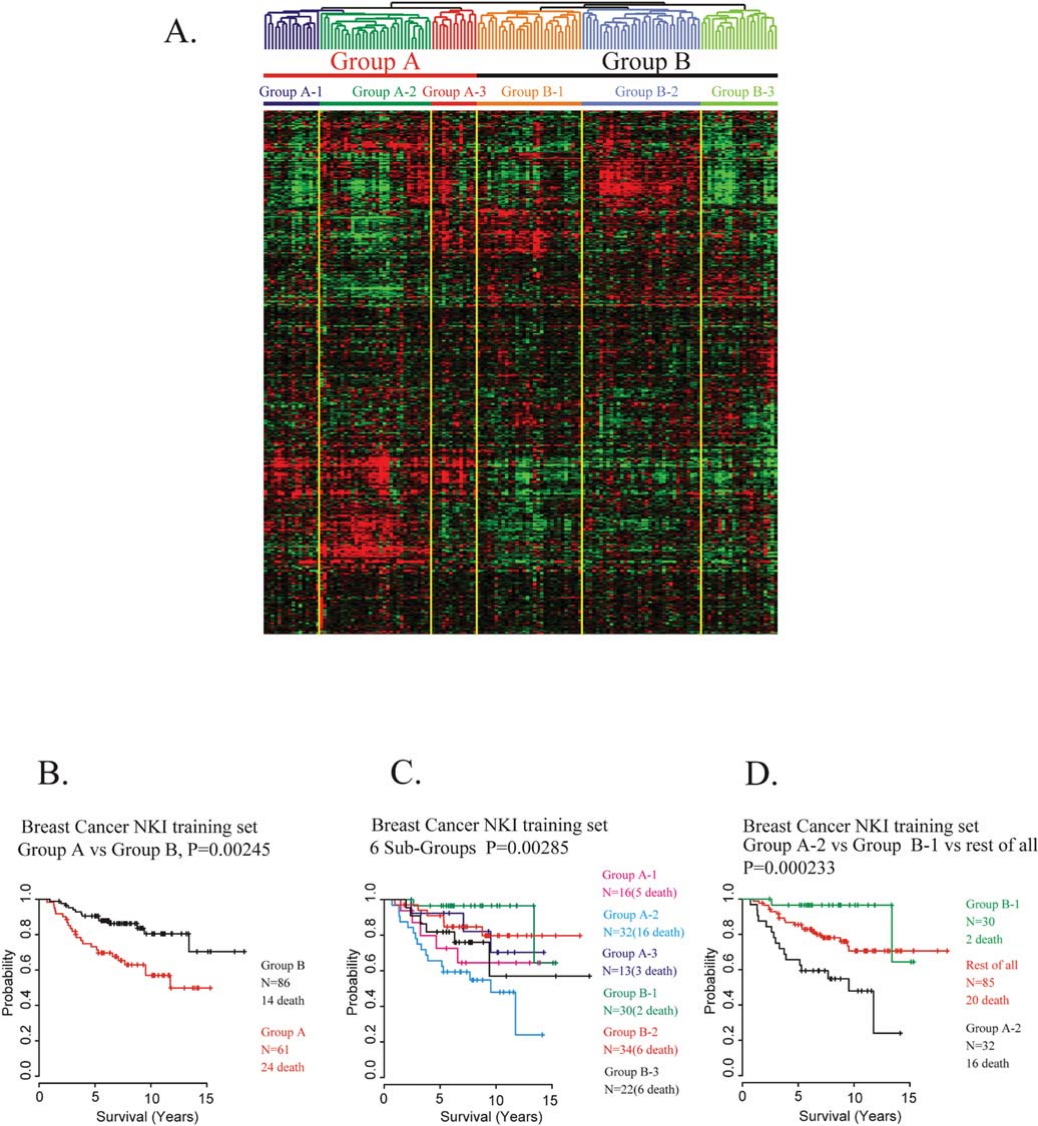
Construction of human breast cancer patients' prognosis prediction models and evaluation of outcomes **A.** Unsupervised cluster analysis of NKI training data set (147 samples). It generated two main clusters and six sub-clusters of patients. **B.** Kaplan-Meier survival analysis of the two main clusters (Group A and Group B). **C.** Kaplan-Meier survival analysis of the six subclusters (Group A1–A3 and Group B1–B3). **D.** Kaplan-Meier survival analysis of two sub-clusters (Group A2 and Group B1) showing WORST and BEST prognosis and one group that includes all the others.

Having demonstrated that our mouse PCT-TC signature can reflect different prognostic features in human patients with three different types of cancer, we focused the application of our PCT-TC expression model to attempt to stratify prognostic subgroups in the well documented human breast cancer microarray data from Netherlands Cancer Institute (NKI) [2], [3]. Unsupervised hierarchical cluster analysis of half of these samples (the training set) using 460 orthologous genes corresponding to our mouse PCT-TC signature revealed two main clusters (Groups A and B), and each cluster was composed of three sub-clusters (A1, A2, A3 and B1, B2, B3, Fig. 2A).

Survival analysis of each subgroup by Kaplan-Meier plots with log-rank test showed significant differences between the two main clusters (Fig. 2B). The patients in the group with the better prognosis (Group B) had an overall 85% 10-year survival rate and a 90.7% 5-year survival rate while the group with poorer prognosis (Group A) had a 62.3% 10-year survival rate and a 73.8% 5-year survival rate. The difference was significant with a p value of 0.00245.

Kaplan-Meier plot and log-rank test was also performed using the 6 different subgroups of patients, three from each of the above-studied groups A and B. The log-rank test revealed that these six subgroups also had significant differences in survival, with a p value of 0.00285 (Fig. 2C). The Kaplan-Meier plot showed the most dramatic survival differences were between B1 [hereinafter designated as BEST prognosis subgroup with 96.7% 10-year survival rate (the overall 5-year survival rate was the same 96.7%)] and A2 (designated as WORST prognosis subgroup with 53.1% of survival rate in 10 years and 65.6% in 5 years). The other 4 subgroups exhibited less significant differences and were combined into a single intermediate subgroup (Fig. 2D). These 3 groups had very significant differences in survival (p = 0.000233).

**Figure 3:**
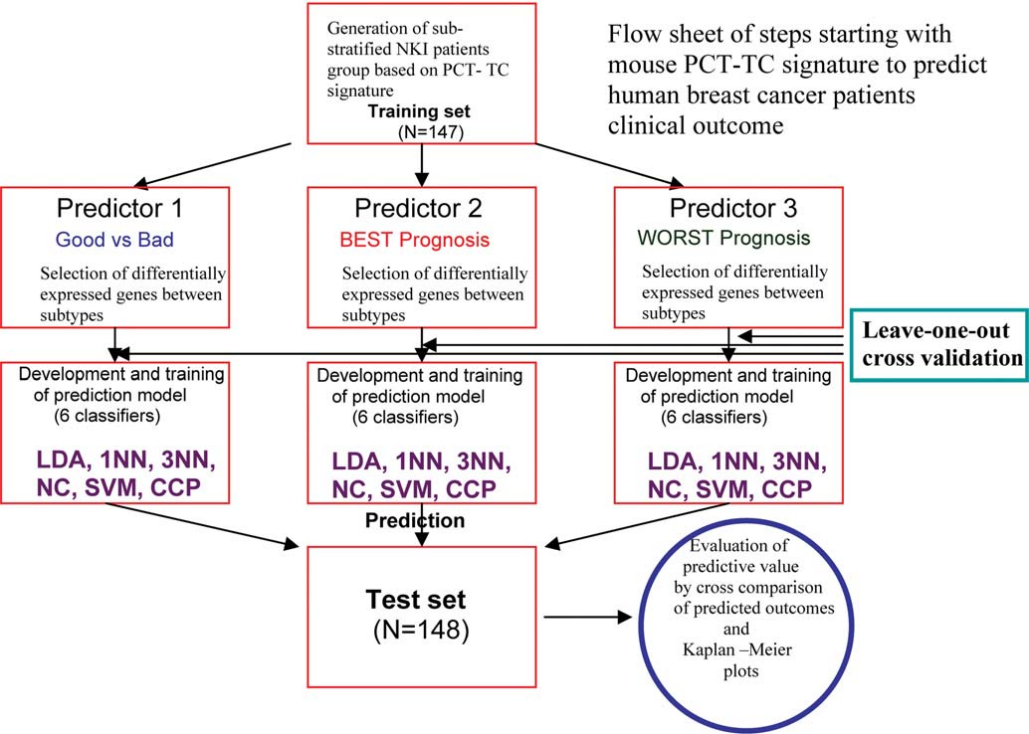
Overview of strategy for the construction of prediction models and evaluation of outcomes. Based on the unsupervised cluster analysis results, 3 independent prediction models are generated.

This part of the study suggested that our PCT-TC signature may indeed have the potential to provide a novel prognostic model that can predict breast cancer patients' prognosis more precisely than models published heretofore. To refine this model further and to sub-stratify the prediction of outcome, we applied 6 different class prediction algorithms [Compound Covariate Predictor (CCP), Linear Discriminator Analysis (LDA), One Nearest Neighbor (1NN), Three Nearest Neighbor (3NN), Nearest Centroid (NC), and Support Vector Machine (SVM)], which compares two groups at a time, to the NKI microarray data in three independent prediction models for the prediction of patients subtype as good prognosis vs. bad prognosis (predictor 1), BEST prognosis vs. not BEST (predictor 2), and WORST prognosis vs. not WORST (predictor 3). All 6 algorithms yielded very similar results, showing the reliability and robustness of our approach. However, we felt that including all 6, followed by vote taking would overcome any weakness in any single prediction method. Figure 3 presents a schematic overview of this strategy for the construction of prediction models and evaluation of outcomes.

**Figure 4:**
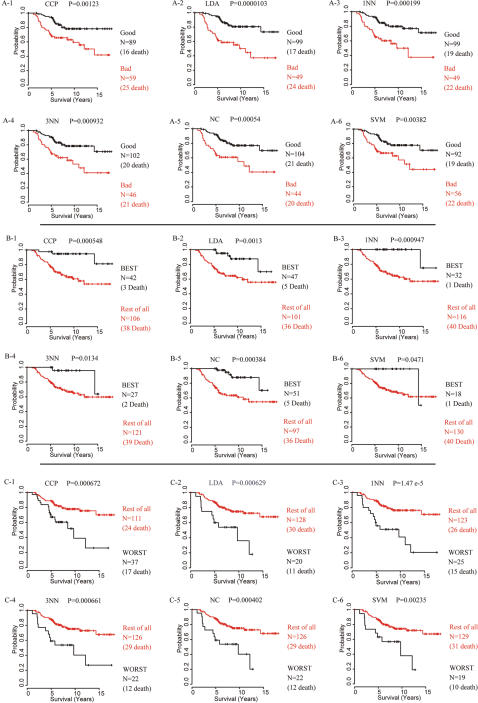
Kaplan-Meier plots of overall survival with NKI validation set predicted by six different prediction algorithms in 3 independent prediction models. **A 1–6.** Group B (with good prognosis) vs. the rest (Group A, with bad prognosis) (Predictor 1). **B 1–6.** Subgroup B1 (BEST prognosis) vs. the rest of all (predictor 2). **C 1–6.** Subgroup A2 (WORST prognosis) vs. the rest of all (predictor 3). The differences between groups were significant in log rank test, with p value indicated above each plot.

The large numbers of human genes in the Agilent microarray that were most differentially expressed between the three different pairs of prognostic subtypes (Good vs. Bad, BEST vs. not BEST, and WORST vs. not WORST) in the training set, were selected independently (two-sample t-test, p value <0.001) and applied as classifiers that estimate the probability of a particular breast cancer patient belonging to one of these specific subtypes using the above-mentioned six different types of prediction methods (CCP, LDA, 1NN, 3NN, NC, and SVM) (see Table S2). When applied to a different group of 148 NKI breast cancer patients as a validation set, all six prediction methods produced consistent patterns. All Kaplan-Meier plots in the test set showed significant differences in survival of patients with specific subtypes when independently analyzed by these six prediction algorithms (Fig. 4). These results demonstrated not only a strong association of gene expression pattern with the survival of the patients, but also a robust reproducibility of these gene expression-based predictors. It is interesting to note that B3 and B6 in this figure seem to pick a set of almost perfect survivors, although these BEST groups are much smaller than that in the other prediction algorithms, presumably due to stricter selection criteria.

### Generation and validation of the four distinct subgroups of human breast cancer

**Figure 5:**
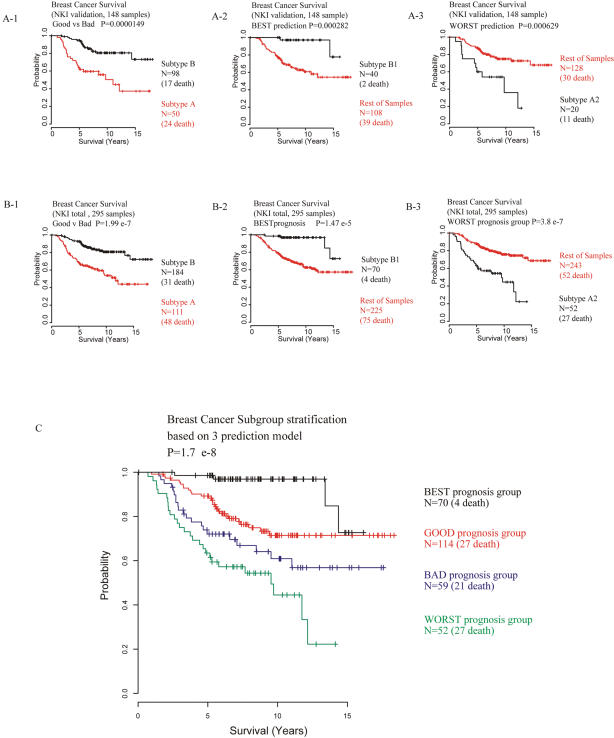
Defining four distinct survival subgroups of human breast cancer **A.** Predicted outcomes in NKI test set (148 patients). Kaplan-Meier plot for the representative groups for 6 different prediction algorithms. If a sample was predicted to belong to the test class (black lines) 3 or more times in the 6 different prediction methods, it was assigned to that group. Otherwise that patient/sample remained in “rest of all” (red lines). There were no 3:3 ties for predictor 1. For predictors 2 and 3, ties were assigned to BEST and WORST, respectively. **B.** Predicted outcomes for combined NKI data sets (295 total patients). Kaplan Meier plots of overall survival of two independent groups identified with two independent analyses (unsupervised clustering in training data set and class prediction in validation set). **C.** Kaplan-Meier plot of four independent prognostic subtypes generated with the NKI data set. Four independent prognostic subtypes (BEST, GOOD, BAD, and WORST) are assigned as follows. Samples that fell into the Group B (good prognosis group) with predictor 1 (Good vs. Bad) but not assigned to the BEST prognosis group with predictor 2 (BEST vs. all the rest) were assigned to an intermediate group designated GOOD. Similarly, samples that did not fall into Group B (i.e., those that belonged to Group A, the bad prognosis group) with predictor 1 (Good vs. Bad) but not assigned to the WORST prognosis group in predictor 3 (WORST vs. all the rest) were assigned to an intermediate group designated BAD.

After we applied these six prediction algorithms to each of the three class-prediction steps, each patient was assigned to one of two possible groups at each of the three stages, as follows. If samples were classified into a particular class three or more times in the six different prediction methods specified above, it was assigned to that group; otherwise this patient was assigned to the other group. Each member of the groups fit satisfactorily onto similar Kaplan-Meier plots generated with the 6 independent prediction methods (Fig. 5 A1–A3). When the 147 patients in the training set were combined with the 148 patients in the validation set and analyzed in this manner as a single group of 295 patients, similar results were obtained, indicating the homogenous character of the clinical outcomes in the same groups of patients in the training set and the validation set (Fig. 5 B1–B3).

Based on these three sequential stages of class prediction, samples were assigned into four independent prognostic subtypes (BEST, GOOD, BAD, and WORST) as follows. Samples that were assigned to the Good prognosis group in prediction step 1 but not assigned to the BEST prognosis group in prediction step 2 [BEST vs. not BEST] were assigned to an intermediate group designated GOOD. Similarly, samples that did not fall into Good prognosis group in prediction step 1 (Good vs. Bad) and not assigned to the WORST prognosis group in prediction step 3 (WORST vs. not WORST) were assigned to an intermediate group designated BAD. Kaplan-Meier Survival analysis and log-rank test were performed with these four independent subtypes of patients (Fig. 5C), and differences among them were visually apparent. These differences in patient survival were significant (p = 1.7×10^−8^) by the log-rank test. These findings strongly support the view that the four subgroups of human breast cancer assigned by the PCT-TC signature have distinct patterns of gene expression. These differences may reflect significant differences in the mechanism of malignant transformation.

### Statistical evaluation of PCT-TC-based prediction model in human breast cancer

To achieve an independent evaluation of the statistical strength and the prognostic value of our PCT-TC signature-based prediction model in human breast cancer, we applied univariate and multivariate analysis to commonly accepted clinical and pathologic risk factors for human breast cancer progression (see Table S3). The BEST and WORST prognosis predicted groups showed strong association with overall survival in univariate Cox proportional hazards analysis. Multivariate analyses that included all relevant pathological variables, and the predicted subtypes revealed that BEST prognosis group prediction was significantly different from the rest of the prognosis groups, independent of ER status and clinico-pathological features of the tumors. This suggests that its predictive potential has real clinical utility, and the close examination of the genes in this signature might also provide better mechanistic understanding of breast cancer progression.

### Comparison of the results from our model of prognosis prediction with previously defined clinical index and gene signatures

The predicted prognostic subtypes based on our three-stage class-prediction method showed strong association with the status of ER expression, histo-pathological grade [14], 70-gene signature prediction [2], [3], core serum-response signature [15], [16] and ERBB2 signature [17] but not with age, tumor size, the number of positive lymph nodes or treatment (Table 1).

**Table 1:**
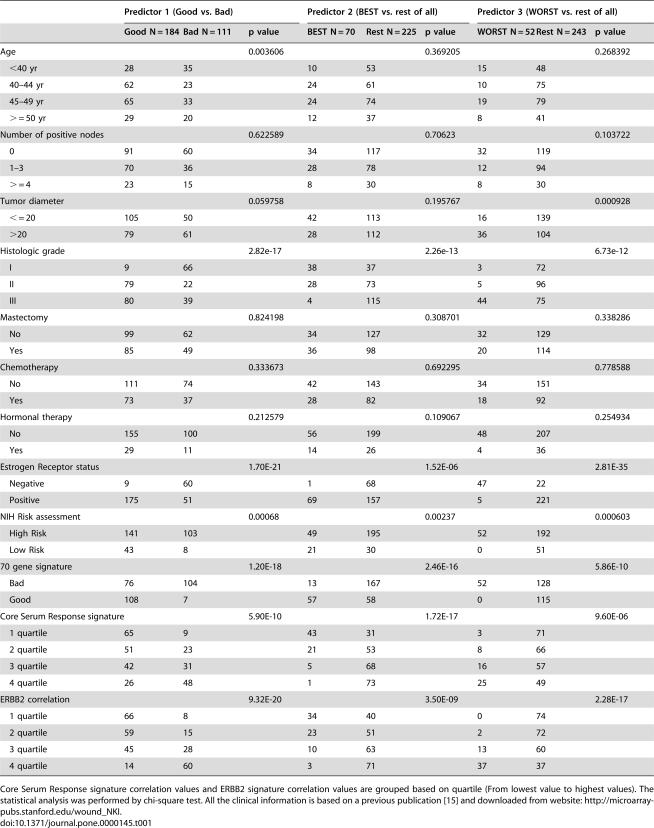
Association study of prognosis prediction model with clinical index using the NKI data set.

	Predictor 1 (Good vs. Bad)			Predictor 2 (BEST vs. rest of all)			Predictor 3 (WORST vs. rest of all)		
	Good N = 184	Bad N = 111	p value	BEST N = 70	Rest N = 225	p value	WORST N = 52	Rest N = 243	p value
Age			0.003606			0.369205			0.268392
<40 yr	28	35		10	53		15	48	
40–44 yr	62	23		24	61		10	75	
45–49 yr	65	33		24	74		19	79	
> = 50 yr	29	20		12	37		8	41	
Number of positive nodes			0.622589			0.70623			0.103722
0	91	60		34	117		32	119	
1–3	70	36		28	78		12	94	
> = 4	23	15		8	30		8	30	
Tumor diameter			0.059758			0.195767			0.000928
< = 20	105	50		42	113		16	139	
>20	79	61		28	112		36	104	
Histologic grade			2.82e-17			2.26e-13			6.73e-12
I	9	66		38	37		3	72	
II	79	22		28	73		5	96	
III	80	39		4	115		44	75	
Mastectomy			0.824198			0.308701			0.338286
No	99	62		34	127		32	129	
Yes	85	49		36	98		20	114	
Chemotherapy			0.333673			0.692295			0.778588
No	111	74		42	143		34	151	
Yes	73	37		28	82		18	92	
Hormonal therapy			0.212579			0.109067			0.254934
No	155	100		56	199		48	207	
Yes	29	11		14	26		4	36	
Estrogen Receptor status			1.70E-21			1.52E-06			2.81E-35
Negative	9	60		1	68		47	22	
Positive	175	51		69	157		5	221	
NIH Risk assessment			0.00068			0.00237			0.000603
High Risk	141	103		49	195		52	192	
Low Risk	43	8		21	30		0	51	
70 gene signature			1.20E-18			2.46E-16			5.86E-10
Bad	76	104		13	167		52	128	
Good	108	7		57	58		0	115	
Core Serum Response signature			5.90E-10			1.72E-17			9.60E-06
1 quartile	65	9		43	31		3	71	
2 quartile	51	23		21	53		8	66	
3 quartile	42	31		5	68		16	57	
4 quartile	26	48		1	73		25	49	
ERBB2 correlation			9.32E-20			3.50E-09			2.28E-17
1 quartile	66	8		34	40		0	74	
2 quartile	59	15		23	51		2	72	
3 quartile	45	28		10	63		13	60	
4 quartile	14	60		3	71		37	37	

Core Serum Response signature correlation values and ERBB2 signature correlation values are grouped based on quartile (From lowest value to highest values). The statistical analysis was performed by chi-square test. All the clinical information is based on a previous publication [15] and downloaded from website: http://microarray-pubs.stanford.edu/wound_NKI.

### Estrogen receptor status and PCT-TC stratification

**Figure 6:**
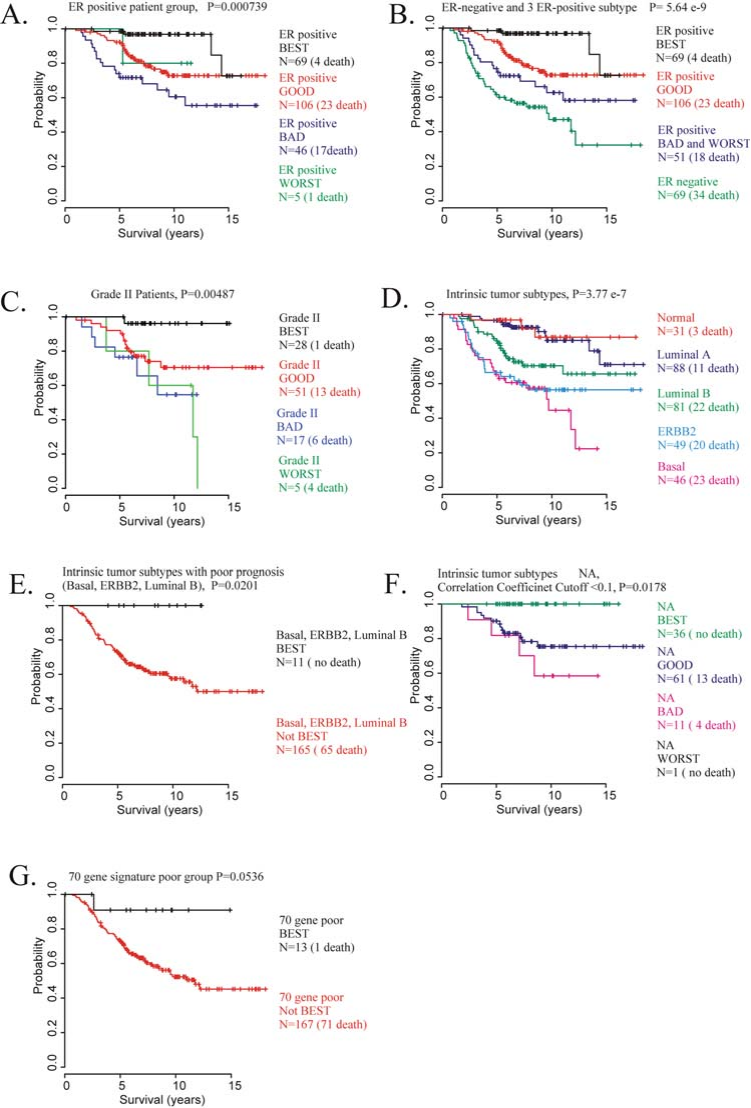
Prediction of breast cancer patients' outcomes based on a combination of gene expression and other criteria The outcome groups previously assigned in the literature based on various criteria (ER status [5], [9], pathological tumor grade [6], intrinsic-sub type [14], [17] and 70-gene signature [2], [3]) were reassessed and further stratified using our prediction system. **A.** Kaplan-Meier plot of ER-positive patients stratified by 3 independent prediction steps. Estrogen receptor-positive patients in the NKI data set were further stratified into the BEST prognosis group (69 patients, 4 deaths), GOOD prognosis group (106 patients, 23 deaths), BAD prognosis group (46 patients, 17 deaths) and WORST prognosis groups (5 patients, 1 death). **B.** Kaplan-Meier plots of survival analysis of ER-negative groups and 3 ER-positive groups that were further stratified by our 3-step prediction analysis. **C.** Kaplan-Meier plot for patients with grade II tumors after further stratification with 3 independent prediction steps. 28 patients were assigned to the BEST prognosis subtype, showing a 96% 15-year survival rate (27 out of 28 patients). The 5 patients assigned to the WORST prognosis subtype had only a 20% (1 of 5 patients) 15-year survival. **D.** Kaplan-Meier plot for intrinsic-sub type. Survival analysis of the complete set of NKI samples (295 patients) previously assigned 5 different breast cancer intrinsic-subtypes (Luminal A, Luminal B, ERBB2, Basal, and Normal Breast-like) by nearest centroid class prediction. **E.** Kaplan-Meier plot for patients with intrinsic-subtypes associated with bad prognosis (Basal, ERBB2+, and Luminal B) after further stratification with our 3-step prediction analysis. This predictor revealed 11 patients that fell into the BEST prognosis group (no deaths within 15 years). **F.** Kaplan-Meier plot for the cell types that could not be assigned (NA) based on correlation coefficients cutting threshold of 0.1. Samples previously not assigned (NA) to any of histological cell types were stratified using our prediction system, revealing subgroups with significantly different clinical outcomes. **G.** Kaplan-Meier plot for the poor prognosis group in the 70-gene-based prediction after further stratification with our 3-step prediction analysis.

It is well accepted that ER-positivity is an indicator of good prognosis in general [5], [9], while ER-negativity usually indicates a poor prognosis, perhaps because these tumors are unlikely to respond to tamoxifen therapy. Our analysis showed that the ER-negative patients (N = 69) constituted a relatively homogenous group with a bad prognosis [47 patients (68%) in the WORST prognosis group and 13 patients (18.8%) in the BAD prognosis group]. On the other hand the ER-positive patients group (N = 226) proved to be much less homogeneous by our PCT-TC analysis, consisting of mainly 3 subtypes (only 5 patients fell into the WORST group): BEST prognosis group (69 patients, 30.5%), GOOD prognosis group (106 patients, 46.9%) and BAD prognosis group (46 patients, 20.3%). When we performed Kaplan-Meier Survival analysis with these sub-stratified groups of ER-positive patients, our prediction system gave a significant improvement of prognostic prediction (log-rank test p = 0.000739) (Fig. 6A). A combined survival analysis of the group of ER-negative patients with the ER-positive patients that had been stratified into 3 subtypes by our PCT-TC-based predictor, defined 4 independent subtypes with significant differences in survival estimates (log-rank test p = 5.64×10^−9^, Fig. 6B).

### Pathologic tumor grading and PCT-TC stratification

In an attempt to improve the prognostic usefulness of histological grading of breast cancer we combined our predicting system with classic clinico-pathological phenotypes [18] for the further stratification of patients, analogous to the approach of Soutiriou et al., [6]. When we stratified the 101 NKI patients with grade-II tumors (intermediate malignancy) using our PCT-TC prediction system, 28 patients were assigned to the BEST prognosis subtype (Fig. 6C), showing a 96% survival rate (27 out of 28 patients survived at least 15 years). This indicates that some patients with very optimistic prognosis can be identified within the patients with “intermediate” grade-II tumors.

### Histological tumor-cell type and PCT-TC stratification

Two very early publications [14], [17] used microarray-based global gene expression analysis to sub-classify breast cancer patients and defined five different breast cancer “intrinsic subtypes”; Luminal A, Luminal B, ERBB2, Basal, and Normal Breast-like. These intrinsic subtypes were associated with significant differences in clinical outcomes. Moreover, it was also reported that those 5 intrinsic-subtypes could also be recognized in a subset of the patients in the NKI reports (117 tumors from young patients), and these subclasses were associated with similar clinical outcomes [19]. When we performed survival analysis of the complete set of the 295-patient set of NKI samples that had been previously assigned to one of the 5 intrinsic-subtypes by nearest centroid classification [19], it confirmed the expected differences in clinical outcomes (log rank test p = 3.77×10^−7^) (Fig. 6D). Applying our PCT-TC analysis, we achieved an even further stratification of 165 breast cancer patients with the histological types that had been associated with a bad prognosis, ERBB2-positive, Basal and Luminal B groups [14], [17]. This analysis separated out a group of patients with a BEST prognosis signature (no deaths in 15 years for these 11 patients) (Fig. 6E).

In addition, however, the correlation coefficients-based assignment of tumors in the initial publication Sorlie et al. [17] generated a large number of patients (109 patients from 295 patients) called “NA” (not assigned) even though very low correlation coefficients cutoff values (<0.1) had been applied. Re-assignment of the NA samples based on our PCT-TC prediction system identified 3 subgroups of patients that had significantly different clinical outcomes (Fig. 6F).

### The 70-gene signature and PCT-TC stratification

In the analysis of the NKI data [2], [3], a 70-gene signature was identified that had a very strong predictive power for a two-way prognosis prediction (good vs. poor). Unfortunately, our analysis showed that this signature was not as strong in prognosis-predicting power when applied to an independent data set, such as that from the Duke University [4] (Fig. S1 ). One contributor to this loss of prediction power was the diminished numbers of predictor genes [only 18 genes (24 probes) of the 70-gene signature in the Agilent microarray set used for the NKI patients' samples were present in the Affymetrix microarray used at Duke].

We attempted a further stratification of these two groups of patients with our PCT-TC strategy from the NKI data set. Analysis of the “poor” prognosis group from the 70-gene signature analysis using our Predictor 2 (BEST vs. not BEST) culled out a BEST prognosis group [13 patients (8% of the “bad” group) with only one death in 15 years; Fig. 6G].

### Prediction of independent human breast cancer patients

To further validate our prediction system, we tested our PCT-TC-derived predictors on two other groups of breast cancer patients whose survival and microarray expression data have been published but which utilized different microarray platforms [a Duke University report, using Affymetrix arrays [4], and a University of North Carolina (UNC) report, using cDNA spotted arrays [5]].

**Figure 7:**
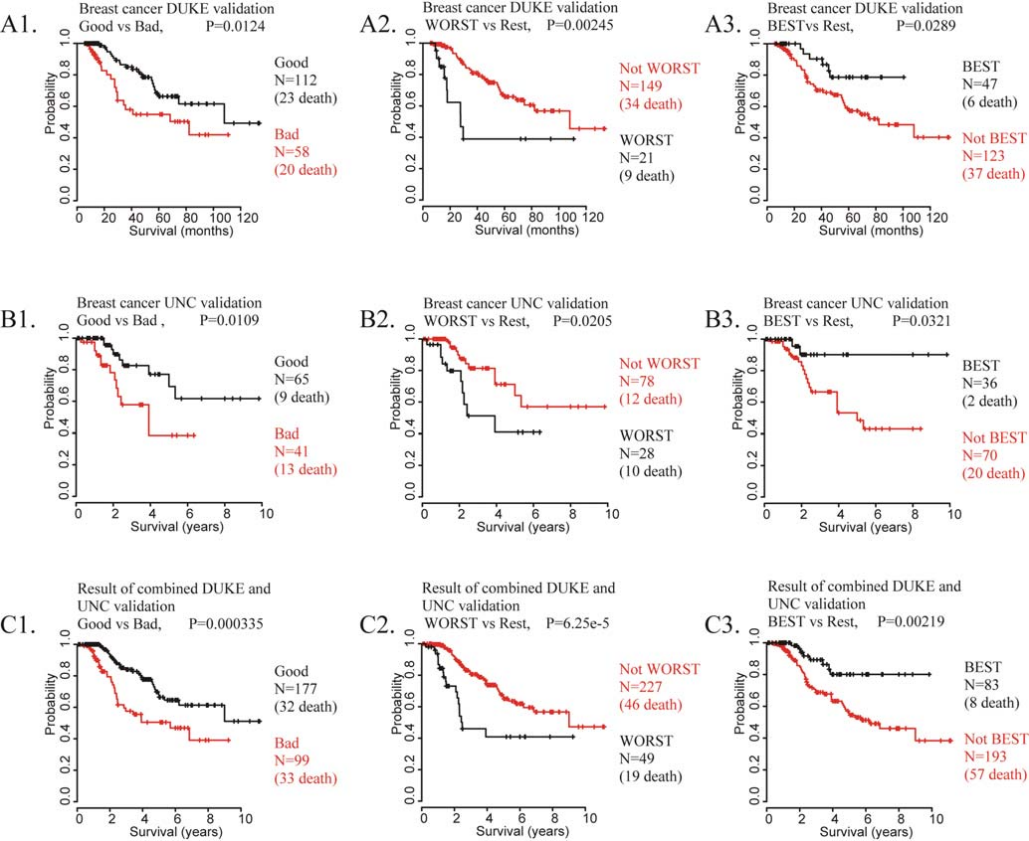
Prediction of independent cohorts of human breast cancer patients The results are shown as the summarized predicted outcomes determined from the results of 6 different prediction algorithms. **A.** Kaplan-Meier plots for the summarized predicted outcomes of Duke University patients [4]. **B.** Kaplan-Meier plots for the summarized predicted outcomes of UNC patients [5]. **C.** Kaplan-Meier plots for the combined predicted outcomes of UNC data and Duke University patients.

Our independent analyses of these two data sets yielded subgroups with significant differences in survival, similar to our findings with the NKI patients. The summarized results of 6 different prediction algorithms for the Duke University testing samples showed patterns similar to the plots for NKI test set of portraits and showed significance in the log-rank tests for each of the three predictor stages: (p = 0.0124 for Good prognosis vs. Bad prognosis, p = 0.0289 for BEST prognosis vs. not BEST, and p = 0.00245 for WORST prognosis group vs. not WORST) (Fig. 7 A1–A3). Analysis of the UNC data with our prediction system also stratified the patients showing patterns similar to the NKI data (p = 0.0109 for Good prognosis vs. Bad prognosis, p = 0.0321 for BEST prognosis vs. not BEST, and p = 0.0205 for WORST prognosis group vs. not WORST) (Fig. 7 B1–B3). Combined analysis of Duke data with UNC data showed outcomes similar to those revealed in the NKI data set (p = 0.000335 for Good prognosis vs. Bad prognosis, p = 0.00219 for BEST prognosis vs. not BEST, and p = 6.25×10^−5^ for WORST prognosis group vs. not WORST) (Fig. 7 C1–C3).

Thus, our method of outcome prediction for two independent groups of patients provided an accurate and precise estimate of clinical outcomes that worked on microarray data sets generated using different microarray platforms and different cohorts of patients treated at different clinical institutions.

### Biological insights into the subtypes of human breast cancer

**Figure 8:**
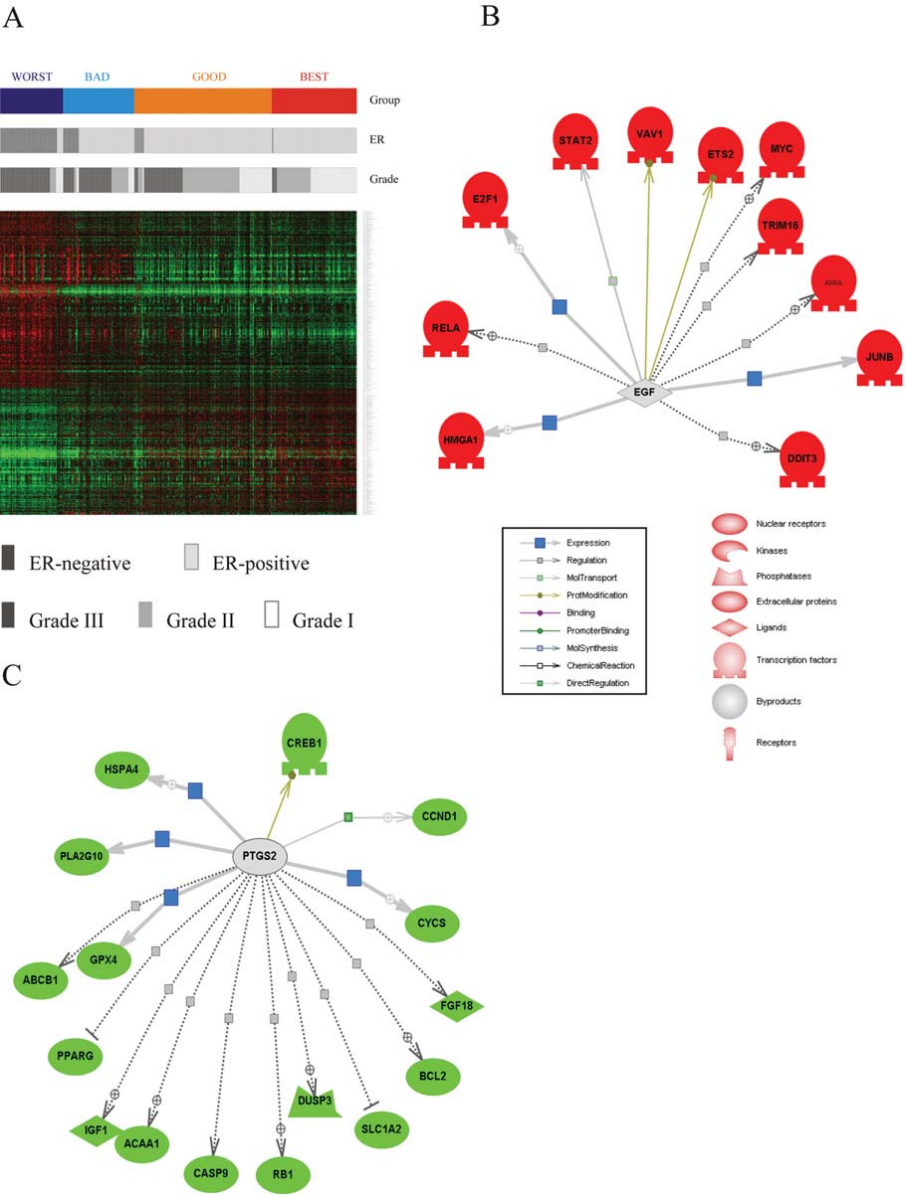
Genes showing significant differences in expression among independent groups (BEST, GOOD, BAD and WORST) **A.** Gene clustering of 295 NKI samples using genes selected by one-way ANOVA class comparisons. A total of 3307 genes that showed significant expression differences (p<1×10^−8^) in a one-way ANOVA analysis were selected. ER expression status and the histo-pathological grade of each tumor sample are shown in grey-scale bars beneath the colored BEST – WORST classification bar. The key to the grey-scale designations is found beneath the heat map. **B.** PathwayAssist^TM^–generated figure showing networks of transcription factors activated by EGF and showing significantly higher expression in tumors from patients in the WORST prognosis group (indicated by red color) compared to the BEST prognosis group. **C.** PathwayAssist^TM^–generated figure showing networks of genes activated by PTGS2 (COX2) and showing significantly lower expression in tumors from patients in the WORST prognosis group (indicated by green color) compared to the BEST prognosis group.

A class comparison of the patients in the four different survival subtypes of the 295 NKI breast cancer patients generated by PCT-TC stratification by one-way ANOVA analysis provided a list of the genes that characterized the different prognostic groups. A total of 3307 genes showed significant differences (p<1×10^−8^) in this analysis (Fig. 8A). This list is too long to be included here, but it included many genes that had been noted in previously reported analyses of gene expression in human breast cancer. General agreement between prognosis subtype and clinical predictors, ER status and histopathological grade can be visually appreciated, although some important exceptions can be seen. Regardless of ER status and histological grade, most of the patients' tumors within a single subtype showed similar gene expression patterns.

Figures 8B and 8C present genetic networks generated by PathwayAssist^TM^ (Ariadne Genomics) analysis of these expression data. Figure 8B shows transcription factors most highly expressed in the WORST subgroup, all of which are activated by EGF. This finding suggests that continuous, spontaneous stimulation of EGFR and Her2 signaling may play central roles in particularly dangerous and metastatic breast cancer tumor types. Figures S2–[Supplementary-material pone.0000145.s003] [Supplementary-material pone.0000145.s004] S5 depict the large number of genes activated by EGF, IFNG, IL4 and CCNA2 that also show increased expression in the WORST group of the NKI patients' tumors, consistent with the previous notion. Of course, these pathways have considerable cross talk or overlap in involved genes. Figure S8 is a Venn diagram that depicts the overlapping genes that are highly expressed in patients with the WORST prognosis and are involved in the EGF, IL4 and IFNG pathways. Figures S6 and S7 depict the large number of genes activated in the WORST group that contribute to the activation of TNF and AKT. This might explain how the anti-apoptotic action of AKT signaling is increased in tumors that fall in the WORST prognosis group and how TNF contributes to the metastasis of the WORST prognosis group.

Figure 8C depicts genes activated by PTGS2 (COX2) that shows significantly lower expression in the WORST prognosis subgroup and higher expression in the BEST subgroup. The higher expression of IGF1, BCL2, and CCND1 in the BEST prognosis group (Fig. 8C) was unanticipated, as these genes are commonly considered to be tumor- promoting genes. The activation of IGF1, BCL2 and CCND1 by PTGS2 might be the prolonged legacy of chronic inflammation in early stages of tumor generation. Following the chronic inflammation stage, genetic or epigenetic changes leading to altered expression of other cytokines, growth factors and oncogenes might take over and be responsible for progression of the tumor.

## Discussion

Even though cancers occur in different organs and involve transformation of many cell types, most cancers share certain basic differences that separate them from normal cellular counterparts [20]. Tumors can evade cell death and bypass mechanisms that normally regulate the cell cycle. Constitutively activated growth factor receptors provide active proliferation signals in many tumors [20], [21]. Such proliferation-promoting genes commonly appear in expression signatures of cancer, and sometimes they can be used to predict clinical outcomes in tumor patients [12], [15], [16]. However, the common accumulation of proliferation signals has made it difficult to use these features to generate highly stratified patient groups with different clinical outcomes and sensitivity to treatment protocols. Hidden beneath the strong proliferation-associated genes may be better clues to variable prognosis, e.g., proteins associated with the ability of cells to live in foreign sites, such as those represented in the PCT-TC signature.

A closer examination of the nature of those genes that provided better stratification of patients may also improve our understanding of the tumorigenic process. In a Gene Ontology (GO) analysis of this signature (see Table S1), many genes involved in angiogenesis (GO:0001525), chemotaxis (GO:0006935), as well as extracellular matrix structural constituents (GO:0005201) and transmembrane receptor tyrosine kinases (GO:0004714) showed significant lower expression in tissue culture versions of PCTs. Most of the genes were previously shown its roles in tumor metastasis and have provided a basis for different prognostic outcomes of human cancer patients.

Molecular phenotypying of breast cancer by Perou et al. [14] delineated four intrinsic-subtypes of breast cancer (Luminal, Basal, ERBB2+, and Normal Epithelial Group) based on 476 genes that showed variable expression in 40 breast cancer samples compared to 20 matched samples that had received doxorubicin treatment. These molecular phenotypes of breast cancer were shown to be conserved in independent analysis of different groups of patients, and they were also associated with significant differences in clinical outcomes that correlated with the tumor cell types.

Another significant advance in the identification of prognostic factors using breast cancer microarray expression analysis was the 70-gene signature generated in axillary lymph node-negative patients by van't Veer et al. [3]. An extended analysis using the 70-gene signature applied to an additional 234 patients [2] documented that this signature-based classification identified classes of patients with significant differences in clinical outcomes more consistently and accurately than any clinical index.

However, it is a challenge for even the best prognostic models when they are tested on different independent patient populations [22], as most of the model parameters for the signature are generally optimized for the original data [23]–[25]. The source of these difficulties may lie in different sizes of patient cohorts or different compositions of patient populations, differences in microarray platforms employed (one dye system vs. two dye system, etc.), and differences in the way the microarray data were processed (normalization, scaling, etc.). Nonetheless, if the predictor used truly reflects basic genetic characteristics relevant to the mechanisms responsible for the different phenotypes (outcomes, prognosis and survival), one would expect these combinations of gene expression to be conserved among all groups of patients with given tumor types. We have shown in detail that our PCT-TC signature-based outcome-predicting model can outperform previous approaches to prognosis prediction for breast cancer patients, and probably for certain other cancers as well. Just how widely this approach can be applied will require further analysis.

Even though genetic signatures may provide the most precise prognostic prediction for individual tumor patients, it is still not widely accepted as an essential component in the choice of therapy. This might be because array-based outcome prediction has been available for only a short period of time, only small numbers of patients and samples have been used for its validation, and because no single approach has emerged with a clear consensus from the clinical/scientific community.

We think that a possible solution to this acceptance problem is to apply multi-faceted models for the prediction of prognosis, which include well-characterized and well-accepted clinical indexes along side newly applied genetic signatures. This approach should minimize the number of false positives and false negatives from tumor staging, ER testing and the potentially subjective interpretation of histo-pathological sections. Adding a microarray analysis step offers the potential of classifying “unclassifiable” tumors or those designated “intermediate” in character. We have shown that there is a considerable heterogeneity in ER-positive populations as well as in patients with grade-II tumors. We feel that application of PCT-TC predictors could supplement existing prediction methods and yield more finely tuned, sub-stratified groups of patients based on molecular genetic similarities. These, then, could be the basis for the understanding of basic mechanisms that might be responsible for different clinical outcomes and provide a means of scientifically predicting prognosis and, if further developed, susceptibility to treatment protocols.

In summary, we generated a novel prognosis prediction model for human breast cancer patients based on a mouse plasma cell tumor tissue culture expression signature. As it is generated based from a data set that originated in a different animal species and a different cell type, it may have side-stepped “over-fitting” problems that often plague models of human cancer patient prognostic prediction. Testing of our prediction model on 3 independent data sets of human breast cancer patients showed similar sub-stratification regardless of microarray type and on patient groups in different clinical institutions.

## Materials and Methods

### Sample Selection and RNA Preparation

A total of 27 samples of RNA were prepared from transplanted mouse PCT tissues and cell lines derived from them. All solid PCT samples used for microarray hybridization had been induced in BALB/c mice by intraperitoneal injections of pristane [26] and transplanted at least once. Some had been accelerated by injection of Abelson murine leukemia virus concomitant with the pristane injection [27]. PCTs were adapted to growth in tissue culture with difficulty, often requiring cycling through pristane-primed mice before sustained growth *in vitro* could be obtained [28]. All tissue cultured cell lines were maintained in RPMI 1620 medium with 10% fetal bovine serum and 10 ng/ml IL-6. All mice were maintained in our conventional colony on the NIH campus under Animal Study Protocol LG-028. Total RNA was prepared from tissue culture pellets or from frozen tissues pulverized by mortar and pestle in liquid nitrogen using extraction in TRIzol (Invitrogen) followed by further purification on RNAeasy columns (Qiagen).

### Microarray Hybridization

Affymetrix U74Av2 microarrays were used for the hybridization of biotin-labeled cDNA probes synthesized from 5 µg of total RNA or 1 µg poly(A)+ RNA using Superscript double–strand cDNA synthesis kit (Invitrogen), Bioarray High Efficiency RNA transcript labeling kits and Mg-catalyzed fragmentation kit (Enzo) according to the manufacturers' instruction. Microarrays were stained with phycoerythrin-streptavidin (Molecular Probes), scanned with Affymetrix GeneChip scanner and analyzed with Affymetrix Microarray Analysis Suite (MAS) version 5.0.

### Statistical Analysis of Microarray Data

BRB ArrayTools Version 3.0 (http://linus.nci.nih.gov./BRB-ArrayTools.html) was used for the analysis of the MAS 5.0 data set. A log base 2 transformation was applied to the data set before arrays were normalized. Each array was normalized using median values of gene expression over the entire array (global normalization). A median array was selected as the reference array for normalization. Class comparison analysis was performed with SAM including an estimation of FDR. Cluster analysis was performed with Cluster and Treeview (http://rana.lbl.gov/EigenSoftware.htm). For the cluster analysis, the log base 2 transformed data were centered to the mean values of each gene's expression.

For the generation of prediction models, samples were divided into two groups as “training set” and “validation set” in such as way that each group had a similar composition of follow-up times since the first diagnosis. Six different prediction methods were applied for the validation of subclasses: LDA, SVM, NC, 1NN, 3NN and CCP. The numbers of genes in the classifiers were optimized to minimize misclassification errors by the leave–one-out cross-validation of the data set. All statistical analyses were performed in R (version 2.0.1). The probabilities of overall survival were estimated according to the Kaplan-Meier method. Significance of difference among subclass was determined by log-rank test.

### Meta-Analysis of Published Data

We worked with several previously published data sets that combined microarray analysis of gene expression in human cancer samples with clinical and laboratory data from the patients at time of tumor discovery and follow-ups and survival analyses of these patients. Five independent groups published valuable sets of data that were publicly available and could be used in our analysis. Mantle cell lymphoma [12] and hepatocellular carcinoma [7] patients were the first groups studied to compare clinical outcomes with global gene expression analyses of mouse PCT-TC orthologs. However, we based most of our studies on the NKI patients in which Agilent two-color oligo microarrays were used for analysis of gene expression [2], [3], because their total patient number, 295, and length of follow-up were the greatest. We also performed meta-analyses on the results generated from 170 breast cancer patients studied at Duke University using Affymetrix single-color microarrays for expression analysis [4], and from 96 patients from the University of North Carolina (UNC) [5] which employed two-color cDNA microarrays. Both data sets provided valuable independent test sets against which we could test our approach for prediction of prognosis. Orthologous genes that were present in the Affymetrix U74Av.2 mouse microarrays and in the human microarrays employed in the previously published studies were selected by using curated mammalian orthology from The Jackson Laboratory. For the analysis of more than one independent data set of breast cancer patients (e.g., NKI data sets and the Duke University data set) each data set was normalized separately and then combined together. Each set was divided into a training set and a test set. Before integrating testing data set with training data set, the expression of each gene was standardized to mean±s.d. of 0±1 independently in both data sets.

### Pathway analysis

Once genes were identified as useful in the stratification of patients' outcomes, we attempted to gain insight into molecular mechanisms that might be involved in generating this hierarchy of patient outcomes. We employed PathwayAssist^TM^ (version 3.0, Ariadne Genomics), as an independent pathway analysis tool to identify connections between differentially expressed genes.

**URL.** The Jackson Laboratory: http://www.informatics.jax.org.

BRB ArrayTools: http://linus.nci.nih.gov/BRB-ArrayTools.html.

NKI data: http://microarray-pubs.stanford.edu/wound_NKI

Duke Univ. data: http://data.cgt.duke.edu/oncogene.php

Univ. of North Carolina (UNC) data: https://genome.unc.edu/

## Supporting Information

Figure S1 70-gene signature-based prognosis prediction of Duke University patients. A. Cluster analysis of patients from Duke University [4] using the 70-gene signature from the NKI data set analysis [2]. Note that only 24 probes in the Duke University Affymetrix microarray platform match the 70 genes from the NKI Agilent microarray used to define the prognostic predictor in the NKI data set. B. Kaplan-Meier survival plot of the two main clusters generated in A.
(5.38 MB TIF)

Figure S2 PathwayAssist^TM^-generated figure showing genes that are activated by EGF and show significantly increased expression in the tumors from patients in the WORST prognosis group compared to those in the BEST prognosis group.
(3.18 MB TIF)

Figure S3 PathwayAssist^TM^-generated figure showing genes that are activated by interferon γ and show significantly increased expression in the tumors from patients in the WORST prognosis group compared to those in the BEST prognosis group.
(5.04 MB TIF)

Figure S4 PathwayAssist^TM^-generated figure showing genes that are activated by IL-4 and show significantly increased expression in the tumors from patients in the WORST prognosis group compared to those in the BEST prognosis group.
(4.07 MB TIF)

Figure S5 PathwayAssist^TM^-generated figure showing genes that are activated by cyclin A2 and show significantly increased expression in the tumors from patients in the WORST prognosis group compared to those in the BEST prognosis group.
(3.58 MB TIF)

Figure S6 PathwayAssist^TM^-generated figure showing genes that are activated by tumor necrosis factor and show significantly increased expression in the tumors from patients in the WORST prognosis group compared to those in the BEST prognosis group.
(4.75 MB TIF)

Figure S7 PathwayAssist^TM^-generated figure showing genes that are activated by AKT1 and show significantly increased expression in the tumors from patients in the WORST prognosis group compared to those in the BEST prognosis group.
(5.10 MB TIF)

Figure S8 Venn diagram that shows genes activated in common by EGF, IFN-γ or IL-4. EGF expressed a total of 115 genes significantly more highly in tumors from the patients with the WORST prognosis group than in patients in the BEST prognosis group. Of these 115 genes, 26 are also activated by IL-4 and highly expressed in BEST tumors, and 16 of these are highly expressed in BEST patients' tumors and activated by EGF, IL-4 and TNF. The genes activated by more that one of these three factors are listed in the overlapping sectors.
(3.27 MB TIF)

Table S1 Functional enrichment analysis of PCT-TC signature genes. The enrichment of each gene set was estimated by calculating the cumulated hypergeometric p values of each biological process provided by Gene Ontology Consortium (www.gene-ontology.org). Gene annotation according to Gene Ontology (GO) terms was downloaded from the NCBI (ftp://ftp.ncbi.nih.gov/gene). In order to obtain representative and significantly enriched terms, the terms that belonged to a GO level higher than 2 and that included at least three genes were considered in our calculation. Statistical significance was determined with a cut-off of p<0.001.
(0.42 MB DOC)

Table S2 Summary of the class prediction results for NKI training set. Genes significantly different between the classes at 0.001 significance level were used for class prediction. 4160 genes were selected as a classifier for Good vs. Bad group and 1651 genes were selected as a classifier for BEST prognosis group. For the prediction of WORST prognosis group 4700 genes were selected as a classifier. Leave-one-out cross-validation method was used to compute misclassification rate based on 100 random permutations.
(0.05 MB DOC)

Table S3 Cox univariate and multivariate analysis of risk factors for death. Parameters showing significance in the Cox proportional hazard model are shown in bold. Age is categorized into two groups based on >45 years old or not. Lymph node status is categorized into two groups, one with one or more tumor cells infiltrated into the lymph node and another group with no tumor cells infiltrated into the lymph node. P value for statistical significance was calculated by log-rank test. T1T2 is categorized based on the diameter of tumor size are >2 cm or not.
(0.05 MB DOC)
