# Supplementary figures and images for: Heterologous Tissue Culture Expression Signature Predicts Human Breast Cancer Prognosis
Source: PLoS One. 2007 Jan 3;2(1):e145. doi: 10.1371/journal.pone.0000145 (PMC1764035; doi:10.1371/journal.pone.0000145)

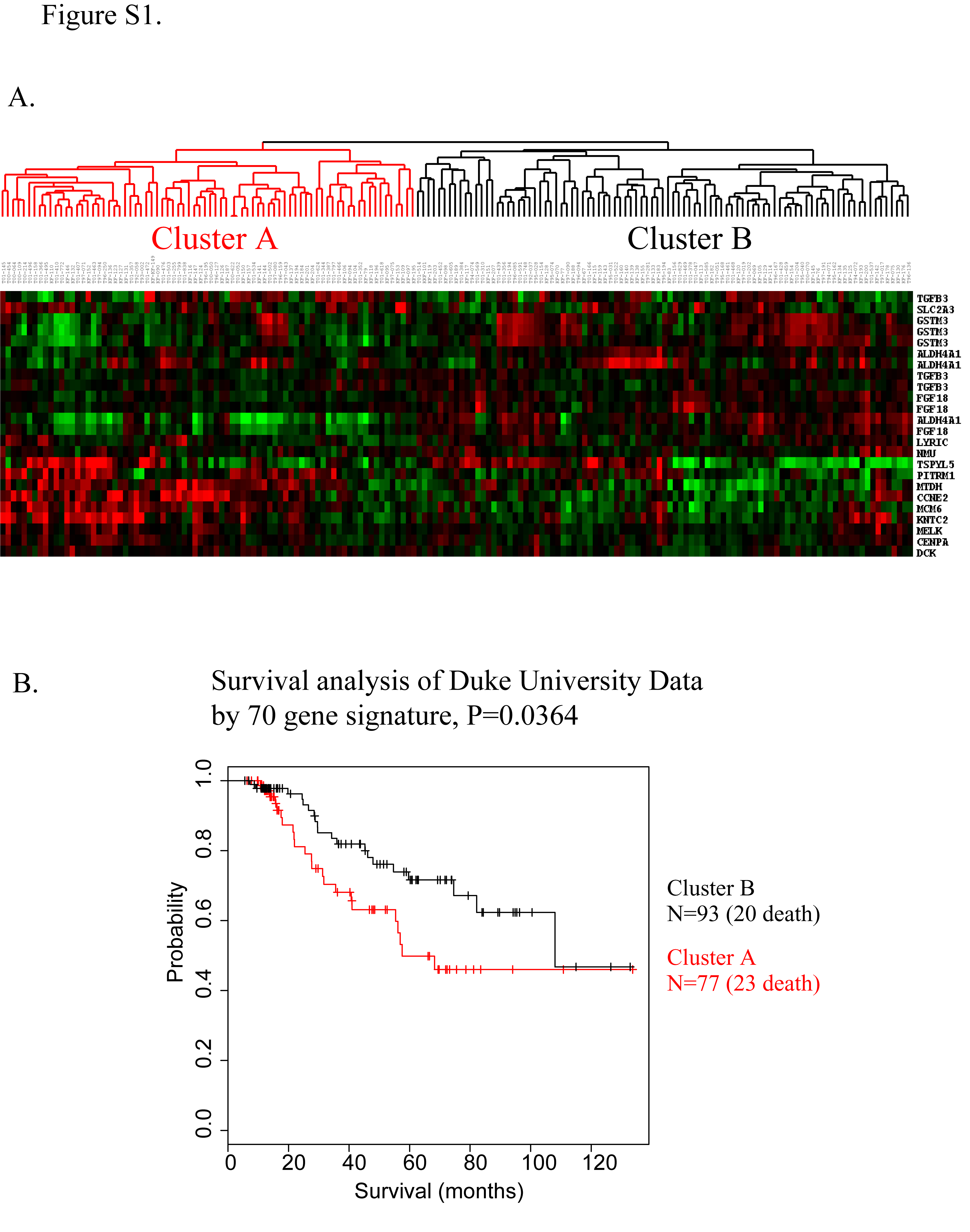

Supplement: Figure S1 — 70-gene signature-based prognosis prediction of Duke University patients. A. Cluster analysis of patients from Duke University [4] using the 70-gene signature from the NKI data set analysis [2]. Note that only 24 probes in the Duke University Affymetrix microarray platform match the 70 genes from the NKI Agilent microarray used to define the prognostic predictor in the NKI data set. B. Kaplan-Meier survival plot of the two main clusters generated in A. (5.38 MB TIF) [file pone.0000145.s001.tif]

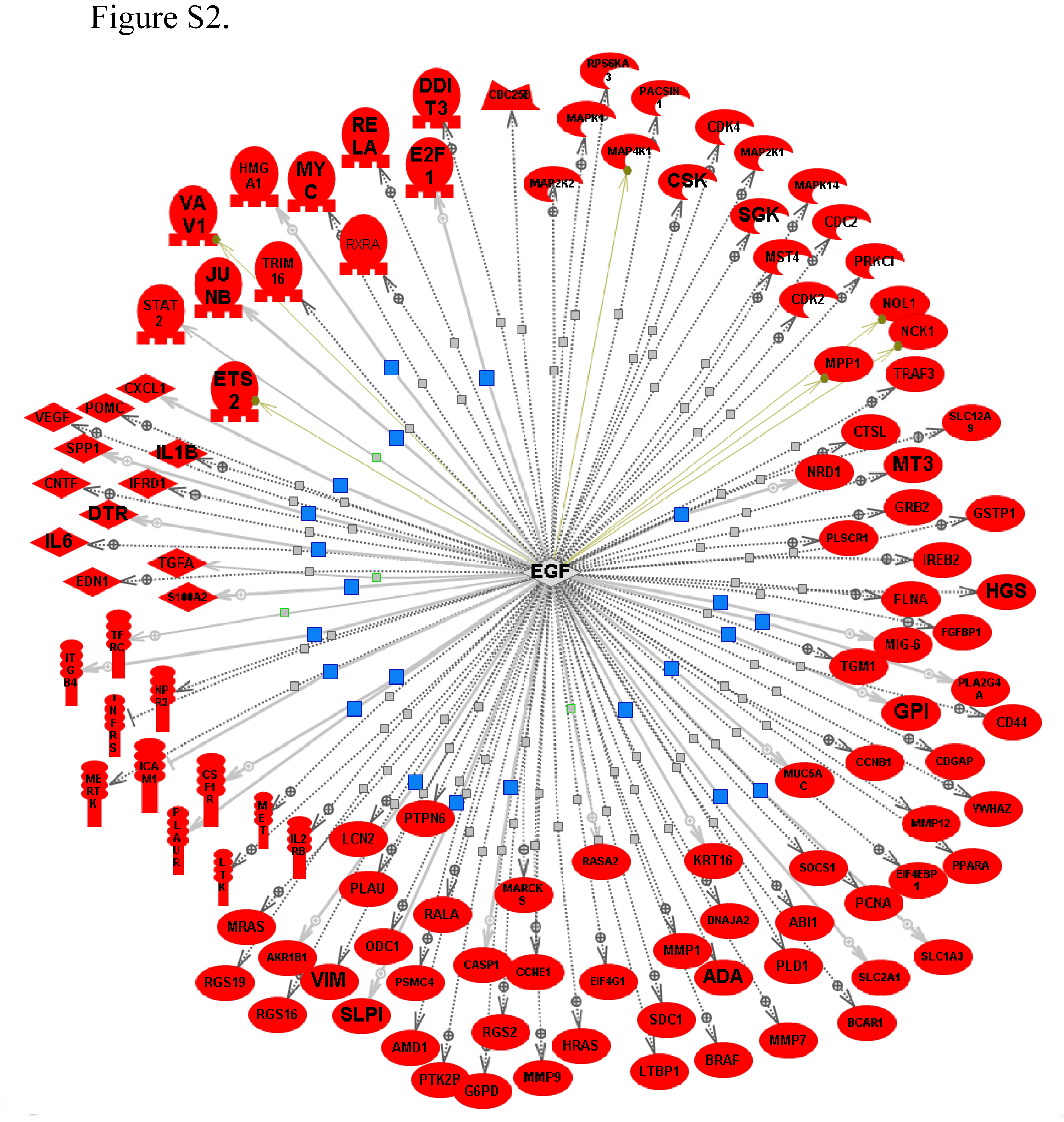

Supplement: Figure S2 — PathwayAssistTM-generated figure showing genes that are activated by EGF and show significantly increased expression in the tumors from patients in the WORST prognosis group compared to those in the BEST prognosis group. (3.18 MB TIF) [file pone.0000145.s002.tif]

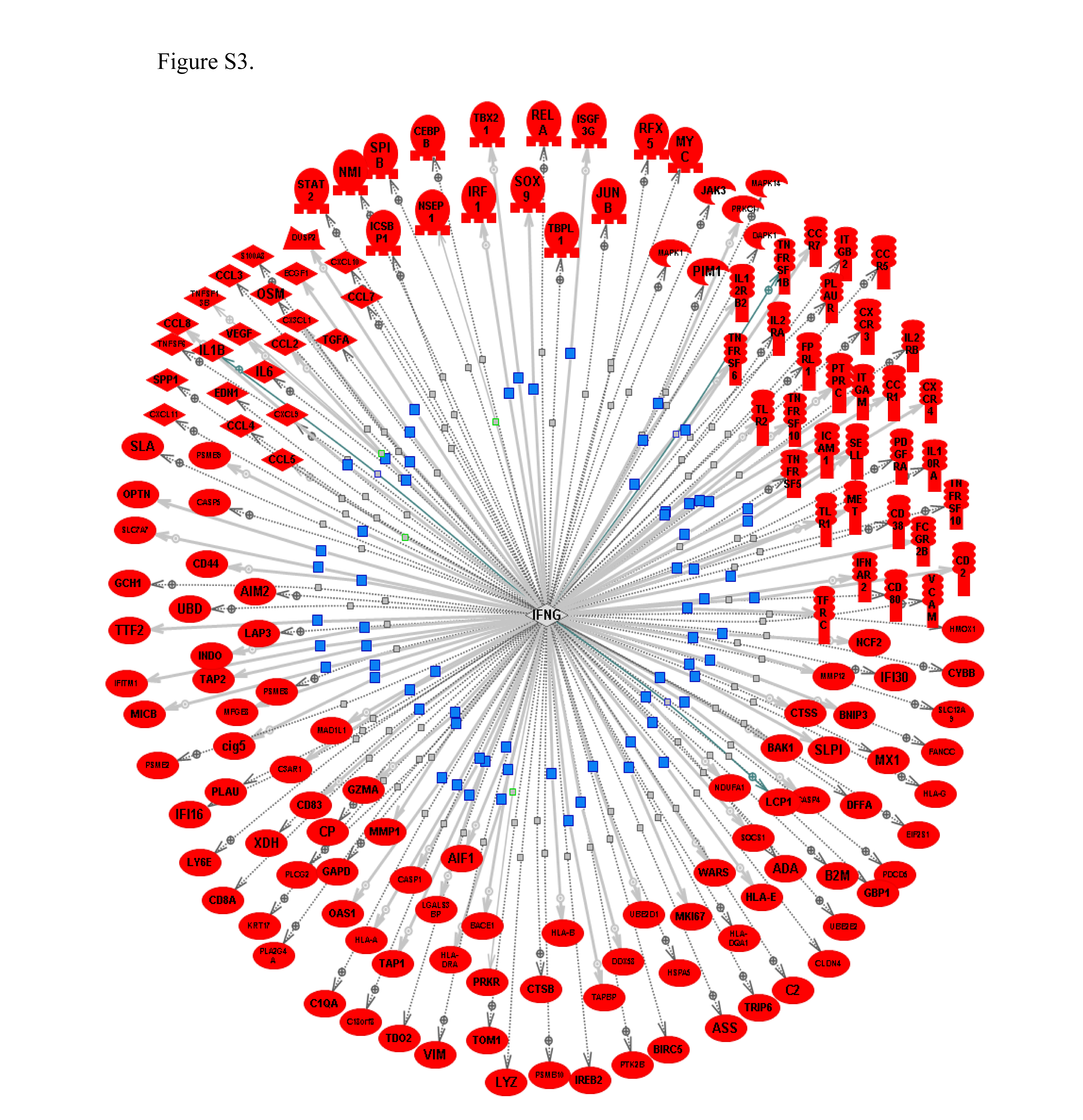

Supplement: Figure S3 — PathwayAssistTM-generated figure showing genes that are activated by interferon γ and show significantly increased expression in the tumors from patients in the WORST prognosis group compared to those in the BEST prognosis group. (5.04 MB TIF) [file pone.0000145.s003.tif]

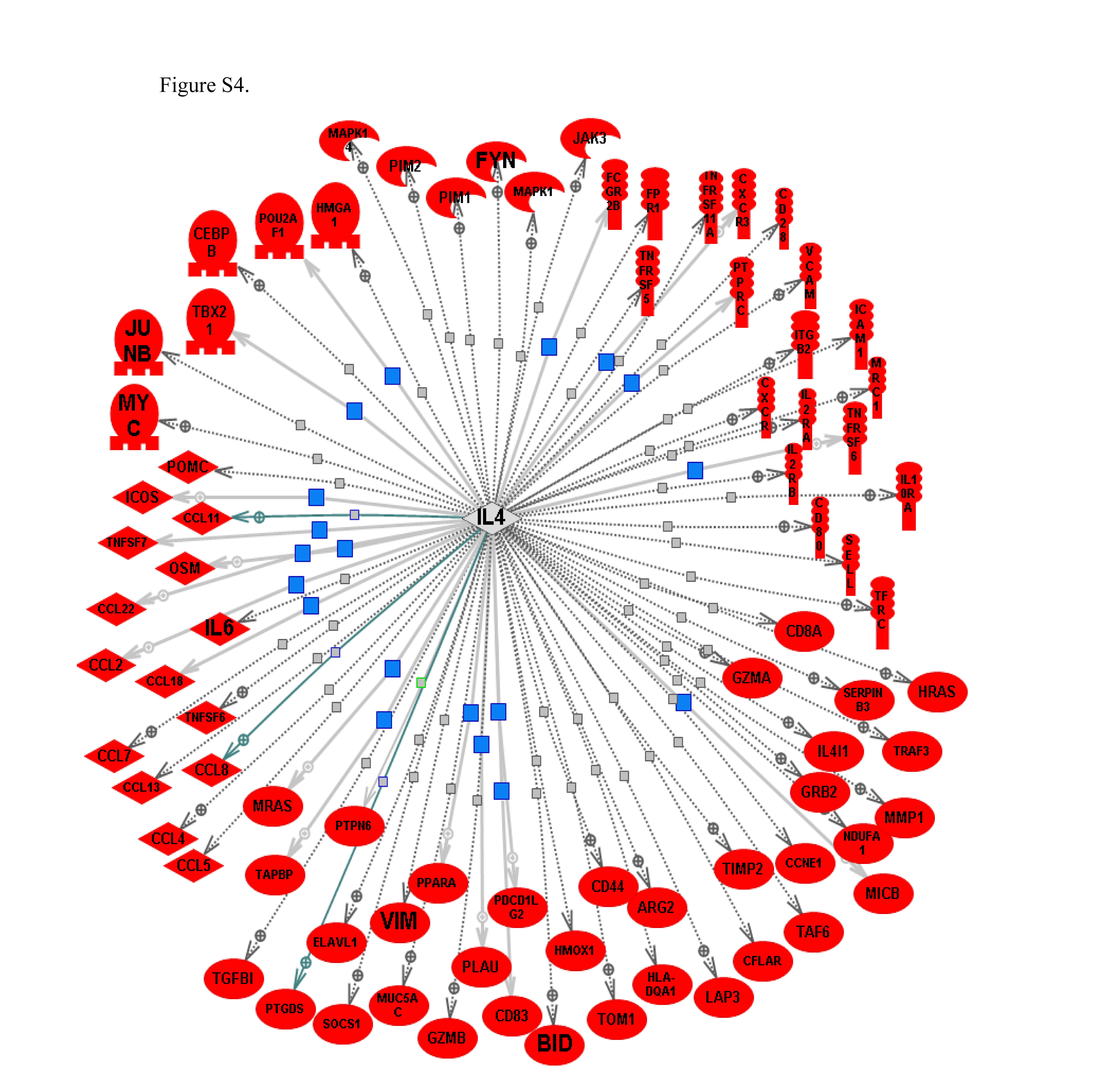

Supplement: Figure S4 — PathwayAssistTM-generated figure showing genes that are activated by IL-4 and show significantly increased expression in the tumors from patients in the WORST prognosis group compared to those in the BEST prognosis group. (4.07 MB TIF) [file pone.0000145.s004.tif]

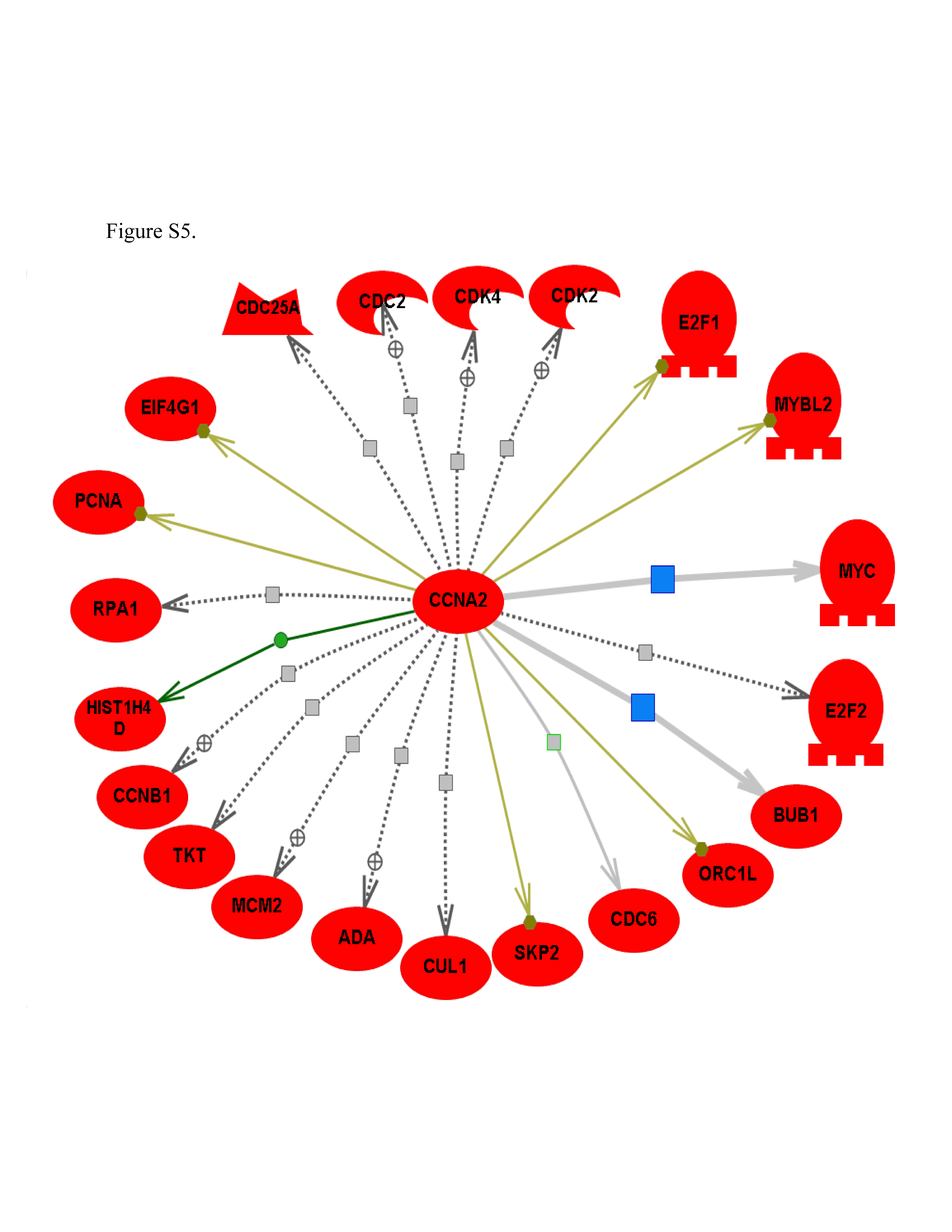

Supplement: Figure S5 — PathwayAssistTM-generated figure showing genes that are activated by cyclin A2 and show significantly increased expression in the tumors from patients in the WORST prognosis group compared to those in the BEST prognosis group. (3.58 MB TIF) [file pone.0000145.s005.tif]

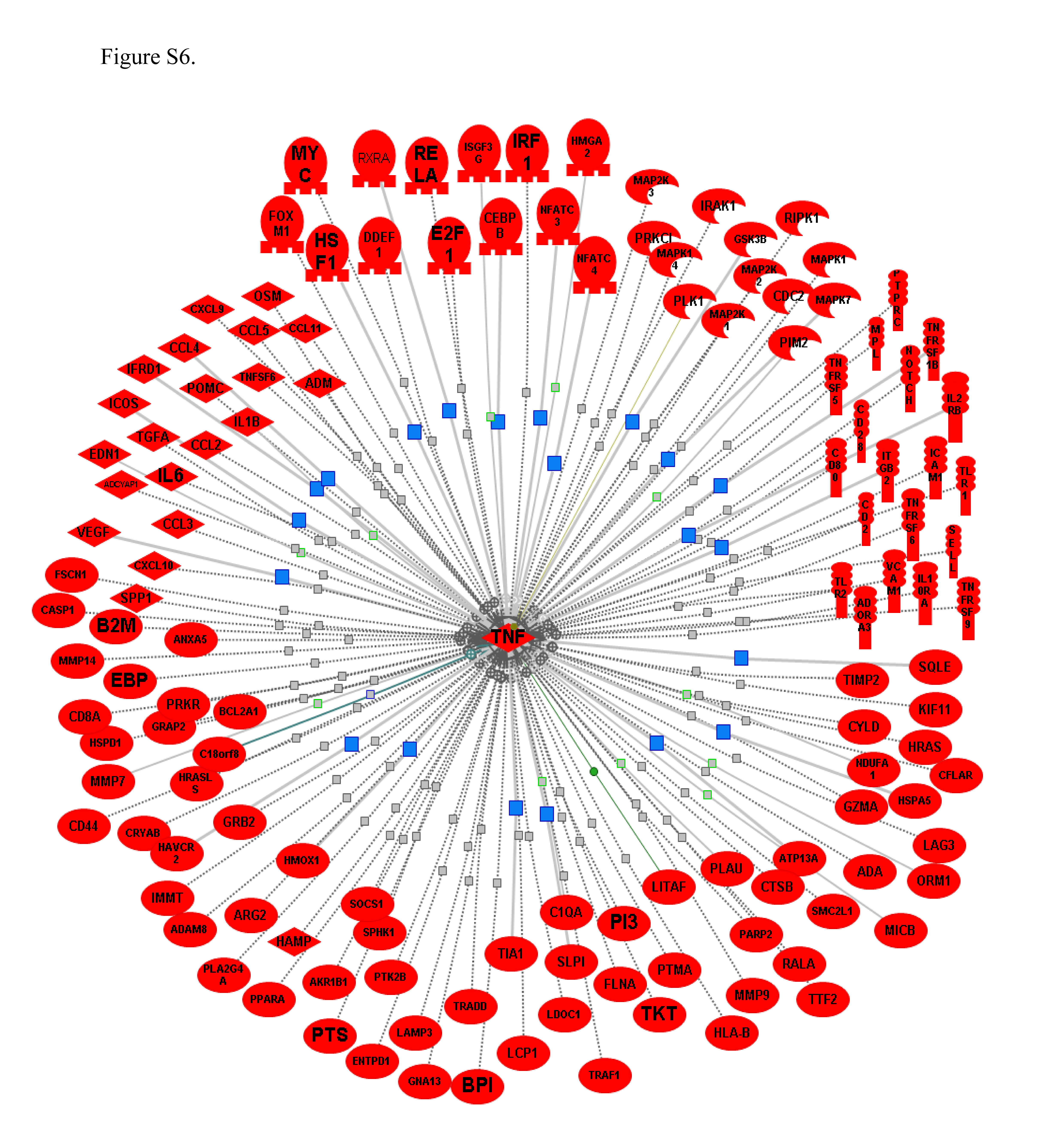

Supplement: Figure S6 — PathwayAssistTM-generated figure showing genes that are activated by tumor necrosis factor and show significantly increased expression in the tumors from patients in the WORST prognosis group compared to those in the BEST prognosis group. (4.75 MB TIF) [file pone.0000145.s006.tif]

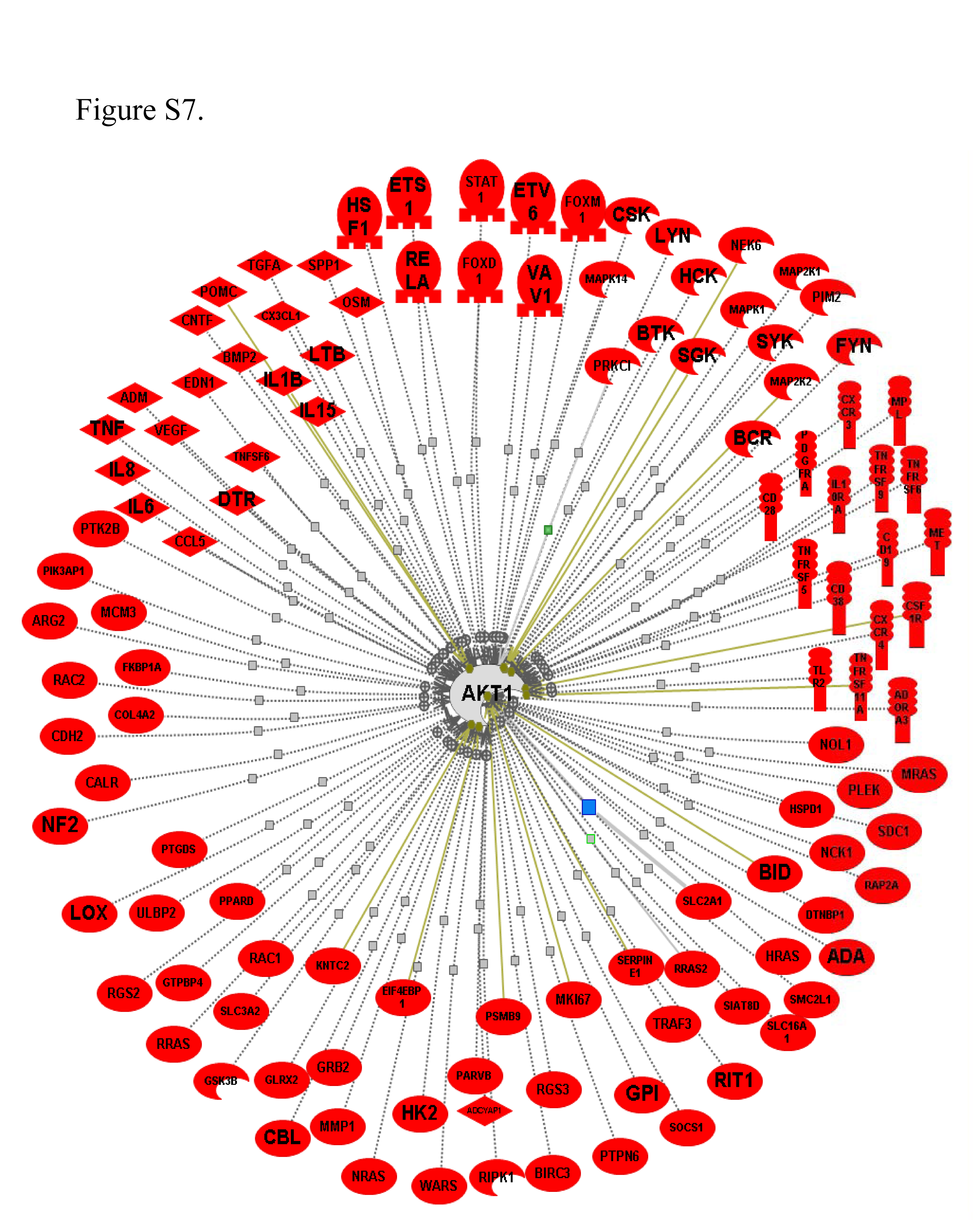

Supplement: Figure S7 — PathwayAssistTM-generated figure showing genes that are activated by AKT1 and show significantly increased expression in the tumors from patients in the WORST prognosis group compared to those in the BEST prognosis group. (5.10 MB TIF) [file pone.0000145.s007.tif]

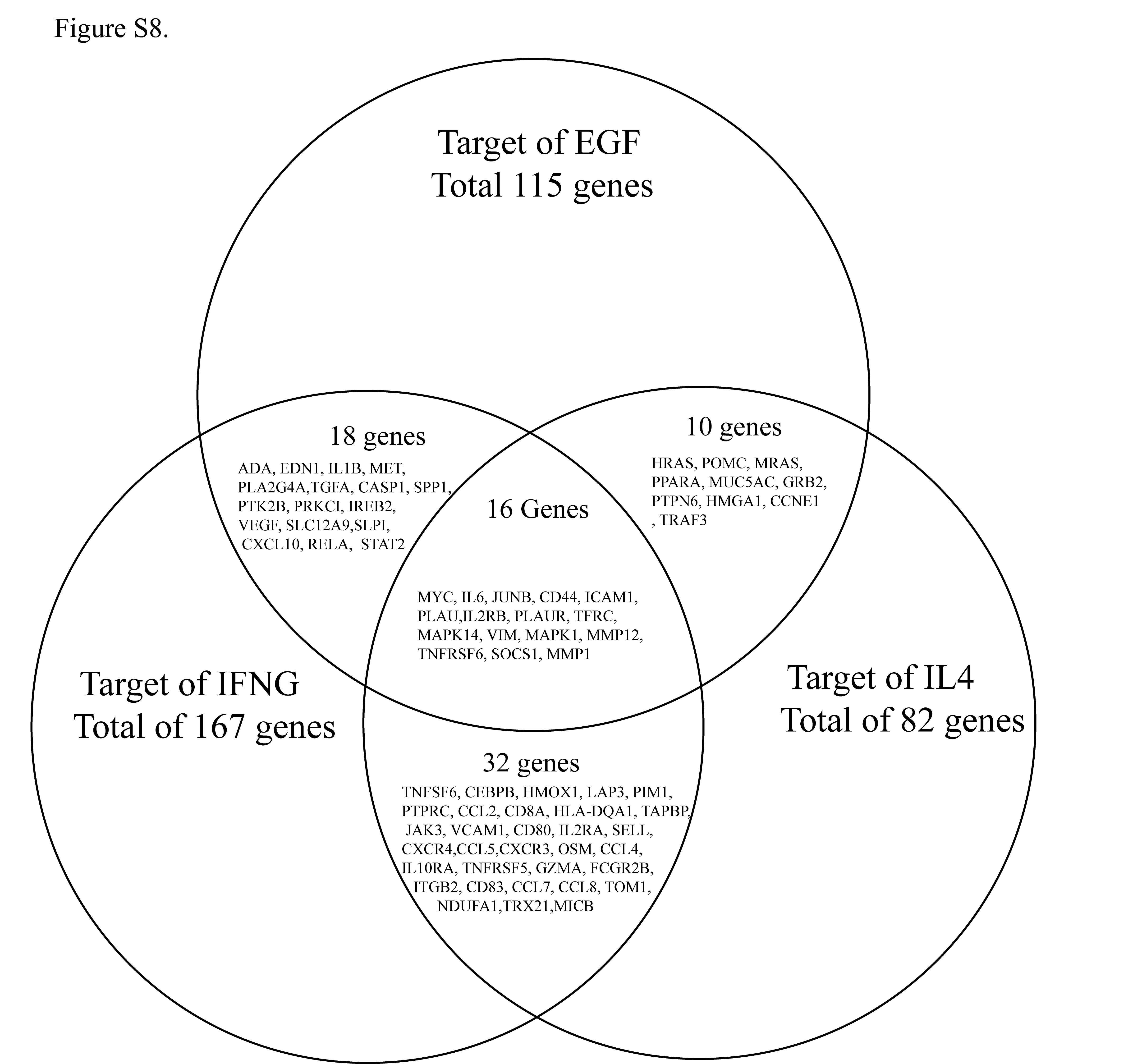

Supplement: Figure S8 — Venn diagram that shows genes activated in common by EGF, IFN-γ or IL-4. EGF expressed a total of 115 genes significantly more highly in tumors from the patients with the WORST prognosis group than in patients in the BEST prognosis group. Of these 115 genes, 26 are also activated by IL-4 and highly expressed in BEST tumors, and 16 of these are highly expressed in BEST patients' tumors and activated by EGF, IL-4 and TNF. The genes activated by more that one of these three factors are listed in the overlapping sectors. (3.27 MB TIF) [file pone.0000145.s008.tif]
