# Supplementary material for: Heterologous Tissue Culture Expression Signature Predicts Human Breast Cancer Prognosis
Source: PLoS One. 2007 Jan 3;2(1):e145. doi: 10.1371/journal.pone.0000145 (PMC1764035; doi:10.1371/journal.pone.0000145)
Supplement: Table S1 — Functional enrichment analysis of PCT-TC signature genes. The enrichment of each gene set was estimated by calculating the cumulated hypergeometric p values of each biological process provided by Gene Ontology Consortium (www.gene-ontology.org). Gene annotation according to Gene Ontology (GO) terms was downloaded from the NCBI (ftp://ftp.ncbi.nih.gov/gene). In order to obtain representative and significantly enriched terms, the terms that belonged to a GO level higher than 2 and that included at least three genes were considered in our calculation. Statistical significance was determined with a cut-off of p<0.001. (0.42 MB DOC) [file pone.0000145.s009.doc]

**Supplementary Table S1. Functional enrichment analysis of PCT-TC signature genes.**

| **Biological Process category** | | | | |
| --- | --- | --- | --- | --- |
| **GO ID** | **P value** | **Name** | Number of genes in category | Number  of genes in the signature |
| GO:0001525 | (p<1 e-06) | angiogenesis | 61 | 14 |
| GO:0006817 | (p<1 e-06) | phosphate transport | 50 | 14 |
| GO:0006955 | (p<1 e-06) | Immune response | 238 | 45 |
| GO:0007155 | (p<1 e-06) | cell adhesion | 358 | 49 |
| GO:0006935 | (p<1 e-06) | chemotaxis | 79 | 18 |
| GO:0006954 | (p=0.000001) | inflammatory response | 96 | 17 |
| GO:0007517 | (p=0.000015) | muscle development | 84 | 14 |
| GO:0048005 | (p=0.000017) | antigen presentation, exogenous peptide antigen | 5 | 4 |
| GO:0007169 | (p=0.000017) | transmembrane receptor protein tyrosine kinase signaling pathway | 54 | 11 |
| GO:0050766 | (p=0.000037) | positive regulation of phagocytosis | 16 | 6 |
| GO:0007166 | (p=0.000132) | cell surface receptor linked signal transduction | 78 | 12 |
| GO:0019884 | (p=0.000149) | antigen presentation, exogenous antigen | 13 | 5 |
| GO:0008285 | (p=0.000231) | Negative regulation of cell proliferation | 49 | 9 |
| GO:0006508 | (p=0.000270) | proteolysis and peptidolysis | 412 | 34 |
| GO:0007010 | (p=0.000327) | cytoskeleton organization and biogenesis | 111 | 14 |
| GO:0050730 | (p=0.000379) | regulation of peptidyl-tyrosine phosphorylation | 9 | 4 |
| GO:0006898 | (p=0.000379) | Receptor mediated endocytosis | 9 | 4 |
| GO:0030154 | (p=0.000392) | cell differentiation | 258 | 24 |
| GO:0006869 | (p=0.000396) | lipid transport | 42 | 8 |
| GO:0006958 | (p=0.000461) | complement activation, classical pathway | 24 | 6 |
| GO:0016126 | (p=0.000829) | sterol biosynthesis | 18 | 5 |
| GO:0008203 | (p=0.000907) | cholesterol metabolism | 27 | 6 |
| GO:0016477 | (p=0.000907) | cell migration | 27 | 6 |
| GO:0007050 | (p=0.000907) | cell cycle arrest | 27 | 6 |

Table S1A. Functional enrichment analysis of Gene Ontology Biological Process category.

| **Molecular Function category** | | | | |
| --- | --- | --- | --- | --- |
| **GO ID** | **P value** | **Name** | Number of genes in category | Number of genes in the signature |
| GO:0004714 | (p<1 e-06) | transmembrane receptor protein tyrosine kinase activity | 29 | 10 |
| GO:0008201 | (p<1 e-06) | heparin binding | 48 | 15 |
| GO:0000166 | (p<1 e-06) | nucleotide binding | 943 | 72 |
| GO:0005509 | (p<1 e-06) | calcium ion binding | 384 | 44 |
| GO:0005515 | (p=0.000001) | protein binding | 2020 | 125 |
| GO:0005021 | (p=0.000002) | vascular endothelial growth factor receptor activity | 7 | 5 |
| GO:0008243 | (p=0.000003) | plasminogen activator activity | 4 | 4 |
| GO:0005201 | (p=0.000005) | extracellular matrix structural constituent | 41 | 10 |
| GO:0005319 | (p=0.000049) | lipid transporter activity | 25 | 7 |
| GO:0004859 | (p=0.000070) | phospholipase inhibitor activity | 3 | 3 |
| GO:0008009 | (p=0.000086) | chemokine activity | 36 | 8 |
| GO:0008191 | (p=0.000090) | metalloendopeptidase inhibitor activity | 7 | 4 |
| GO:0005520 | (p=0.000152) | insulin-like growth factor binding | 21 | 6 |
| GO:0004713 | (p=0.000213) | protein-tyrosine kinase activity | 294 | 26 |
| GO:0004907 | (p=0.000340) | interleukin receptor activity | 24 | 6 |
| GO:0005524 | (p=0.000347) | ATP binding | 1048 | 66 |
| GO:0008233 | (p=0.000359) | peptidase activity | 442 | 34 |
| GO:0004866 | (p=0.000700) | endopeptidase inhibitor activity | 72 | 10 |

Table S1B. Functional enrichment analysis of Gene Ontology Molecular Function category.

Table S1C. Genes present in the Gene Ontology selected by functional enrichment analysis. Among the GOs showing statistically significant differences (p<0.001) in the cumulated hypergeometric analysis of the each biological process provided by Gene Ontology Consortium, the following 10 different Gene Ontology categories were selected to show gene compositions.

| **transmembrane receptor protein tyrosine kinase activity**  GO:0004714  (p< 1 e-06) | | | | | | |
| --- | --- | --- | --- | --- | --- | --- |
| **Affy Id** | **Unigene Id** | **Mean intensity of Solid PCT** | **Mean intensity of TC-PCT** | **Fold difference** | **Description** | **Gene Symbol** |
| 100155_at | Mm.5021 | 662.7 | 204.8 | 3.236 | Discoidin domain receptor family, member 1 | Ddr1 |
| 101842_g_at | Mm.8534 | 214.1 | 84.1 | 2.546 | RIKEN cDNA E230015J15 gene | Egfr |
| 104265_at | Mm.285 | 505.1 | 149.2 | 3.385 | Kinase insert domain protein receptor | Kdr |
| 104417_at | Mm.22574 | 1699.3 | 247 | 6.88 | Colony stimulating factor 1 receptor | Csf1r |
| 160867_at | Mm.3291 | 681.3 | 155.8 | 4.373 | FMS-like tyrosine kinase 4 | Flt4 |
| 161832_r_at | Mm.4146 | 295 | 61.1 | 4.828 | Platelet derived growth factor receptor, beta polypeptide | Pdgfrb |
| 92194_at | Mm.3401 | 58.9 | 17.4 | 3.385 | Proprotein convertase subtilisin/kexin type 5 | Pcsk5 |
| 95079_at | Mm.3464 | 513.1 | 181.4 | 2.829 | FMS-like tyrosine kinase 1 | Flt1 |
| 98453_at | Mm.3464 | 454.3 | 88.2 | 5.151 | FMS-like tyrosine kinase 1 | Flt1 |
| 99956_at | Mm.247073 | 392.2 | 83.1 | 4.72 | Kit oncogene | Kit |

**angiogenesis**

GO:0001525

(p< 1 e-06)

| **Affy Id** | **Unigene Id** | **Mean intensity of Solid PCT** | **Mean intensity of TC-PCT** | **Fold difference** | **Description** | **Gene Symbol** |
| --- | --- | --- | --- | --- | --- | --- |
| 92210_at | Mm.3425 | 503.5 | 69.3 | 7.266 | Angiopoietin 2 | Angpt2 |
| 100569_at  99638_at | Mm.238343 | 4335.2 | 135.9 | 31.9 | Annexin A2 | Anxa2 |
|  | Mm.4352 | 2718.2 | 301.7 | 9.01 | Procollagen, type XVIII, alpha 1 | Col18a1 |
| 100134_at | Mm.225297 | 5767.4 | 719.6 | 8.015 | Endoglin | Eng |
| 102698_at | Mm.1415 | 423.8 | 173.8 | 2.438 | Endothelial PAS domain protein 1 | Epas1 |
| 92365_at | Mm.297978 | 212.4 | 84.2 | 2.523 | C-fos induced growth factor | Figf |
| 93294_at | Mm.1810 | 1606.1 | 16.3 | 98.534 | Connective tissue growth factor | Ctgf |
| 98453_at | Mm.3464 | 454.3 | 88.2 | 5.151 | FMS-like tyrosine kinase 1 | Flt1 |
| 104265_at | Mm.285 | 505.1 | 149.2 | 3.385 | Kinase insert domain protein receptor | Kdr |
| 99993_at | Mm.4487 | 900 | 128.3 | 7.015 | Alanyl (membrane) aminopeptidase | Anpep |
| 95016_at | Mm.271745 | 967.6 | 49.6 | 19.508 | RIKEN cDNA D030005H02 gene | Nrp1 |
| 94932_at | Mm.2675 | 1257.2 | 387.7 | 3.243 | Platelet derived growth factor, alpha | Pdgfa |
| 160489_at | Mm.255332 | 976.2 | 177.8 | 5.49 | Tumor necrosis factor, alpha-induced protein 2 | Tnfaip2 |
| 93917_at | Mm.344820 | 933.4 | 358.8 | 2.601 | Tumor necrosis factor (ligand) superfamily, member 12 | Tnfsf12 |

**inflammatory response**

GO:0006954

(p=0.000001)

| **Affy Id** | **Unigene Id** | **Mean intensity of Solid PCT** | **Mean intensity of TC-PCT** | **Fold differences** | **Description** | **Gene symbol** |
| --- | --- | --- | --- | --- | --- | --- |
| 102025_at | Mm.10116 | 229.1 | 22.1 | 10.367 | Chemokine (C-X-C motif) ligand 13 | Cxcl13 |
| 103486_at | Mm.222830 | 182.4 | 30.3 | 6.02 | Interleukin 1 beta | Il1b |
| 92793_at | Mm.1258 | 1490.1 | 656 | 2.271 | Tumor necrosis factor receptor superfamily, member 1a | Tnfrsf1a |
| 93397_at | Mm.6272 | 534.5 | 87.3 | 6.123 | Chemokine (C-C motif) receptor 2 | Ccr2 |
| 93497_at | Mm.19131 | 2370.6 | 306 | 7.747 | Complement component 3 | C3 |
| 93717_at | Mm.867 | 515.8 | 59.1 | 8.728 | Chemokine (C-C motif) ligand 12 | Ccl12 |
| 93858_at | Mm.877 | 163.4 | 32.5 | 5.028 | Chemokine (C-X-C motif) ligand 10 | Cxcl10 |
| 93871_at | Mm.882 | 767.9 | 150.3 | 5.109 | Interleukin 1 receptor antagonist | Il1rn |
| 95349_g_at | Mm.21013 | 172.1 | 37.6 | 4.577 | Chemokine (C-X-C motif) ligand 1 | Cxcl1 |
| 96886_at | Mm.220821 | 1198.2 | 115.3 | 10.392 | Stabilin 1 | Stab1 |
| 97926_s_at | Mm.3020 | 105.4 | 8.1 | 13.012 | Peroxisome proliferator activated receptor gamma | Pparg |
| 98088_at | Mm.3460 | 2231.2 | 874.7 | 2.551 | CD14 antigen | Cd14 |
| 98304_at | Mm.42146 | 235 | 107.1 | 2.194 | Toll-like receptor 6 | Tlr6 |
| 98406_at | Mm.284248 | 996.6 | 42.1 | 23.672 | Chemokine (C-C motif) ligand 5 | Ccl5 |
| 99413_at | Mm.274927 | 279.7 | 60.6 | 4.616 | Chemokine (C-C motif) receptor 1 | Ccr1 |
| 94761_at |  | 593.1 | 25.9 | 22.9 | chemokine (C-C motif) ligand 7 | Ccl7 |
| 101436_at | Mm.766 | 1076.7 | 29.2 | 36.873 | Chemokine (C-X-C motif) ligand 9 | Cxcl9 |

**chemotaxis**

GO:0006935

(p< 1 e-06)

| **Affy Id** | **Unigene Id** | **Mean intensity of Solid PCT** | **Mean intensity of TC-PCT** | **Fold difference** | **Description** | **Gene symbol** |
| --- | --- | --- | --- | --- | --- | --- |
| 103707_at | Mm.2408 | 624.6 | 117.9 | 5.298 | complement component 3a receptor 1 | C3ar1 |
| 94173_at | Mm.12876 | 344 | 182.1 | 1.889 | chemokine (C-X-C motif) receptor 3 | Cxcr3 |
| 102794_at | Mm.1401 | 2407.5 | 927.8 | 2.595 | chemokine (C-X-C motif) receptor 4 | Cxcr4 |
| 93397_at | Mm.6272 | 534.5 | 87.3 | 6.123 | chemokine (C-C motif) receptor 2 | Ccr2 |
| 161968_f_at | Mm.14302 | 934.1 | 9.5 | 98.326 | chemokine (C-C motif) receptor 5 | Ccr5 |
| 93430_at | Mm.6522 | 321.5 | 17.2 | 18.692 | chemokine orphan receptor 1 | Cmkor1 |
| 99387_at | Mm.56951 | 186.2 | 63.9 | 2.914 | formyl peptide receptor 1 | Fpr1 |
| 93858_at | Mm.877 | 163.4 | 32.5 | 5.028 | chemokine (C-X-C motif) ligand 10 | Cxcl10 |
| 92777_at | Mm.1231 | 302.4 | 63.7 | 4.747 | cysteine rich protein 61 | Cyr61 |
| 99956_at | Mm.247073 | 392.2 | 83.1 | 4.72 | kit oncogene | Kit |
| 93717_at | Mm.867 | 515.8 | 59.1 | 8.728 | chemokine (C-C motif) ligand 12 | Ccl12 |
| 98406_at | Mm.284248 | 996.6 | 42.1 | 23.672 | chemokine (C-C motif) ligand 5 | Ccl5 |
| 92849_at | Mm.137 | 871.7 | 199.5 | 4.369 | chemokine (C-C motif) ligand 6 | Ccl6 |
| 98822_at | Mm.358664 | 1374.8 | 213.2 | 6.448 | interferon, alpha-inducible protein | G1p2 |
| 102025_at | Mm.10116 | 229.1 | 22.1 | 10.367 | chemokine (C-X-C motif) ligand 13 | Cxcl13 |
| 104626_at | Mm.150064 | 504.5 | 240.8 | 2.095 | chemokine-like factor super family 8 | Cklfsf8 |
| 101728_at |  | 1087.9 | 658.5 | 1.652 | complement component 5, receptor 1 | C5r1 |
| 94761_at |  | 593.1 | 25.9 | 22.9 | chemokine (C-C motif) ligand 7 | Ccl7 |

**extracellular matrix structural constituent**

GO:0005201

(p=0.000005)

| **Affy Id** | **Unigene Id** | **Mean inetensity of Solid PCT** | **Mean intensity of TC-PCT** | **Fold difference** | **Description** | **Gene symbol** |
| --- | --- | --- | --- | --- | --- | --- |
| 99638_at | Mm.4352 | 2718.2 | 301.7 | 9.01 | Procollagen, type XVIII, alpha 1 | Col18a1 |
| 98331_at | Mm.249555 | 669 | 17.4 | 38.448 | Procollagen, type III, alpha 1 | Col3a1 |
| 101093_at | Mm.738 | 8683.1 | 77.9 | 111.465 | Procollagen, type IV, alpha 1 | Col4a1 |
| 101039_at | Mm.181021 | 7148.9 | 247.9 | 28.838 | Procollagen, type IV, alpha 2 | Col4a2 |
| 92567_at | Mm.10299 | 6237.8 | 71 | 87.856 | Procollagen, type V, alpha 2 | Col5a2 |
| 95493_at | Mm.2509 | 3647.9 | 669.3 | 5.45 | Procollagen, type VI, alpha 1 | Col6a1 |
| 101130_at | Mm.277792 | 9680.2 | 43.8 | 221.009 | Procollagen, type I, alpha 2 | Col1a2 |
| 92836_at | Mm.275320 | 762.3 | 188.3 | 4.048 | Elastin | Eln |
| 161793_at | Mm.258065 | 778.5 | 179.8 | 4.33 | Laminin, alpha 4 | Lama4 |
| 93472_at | Mm.7281 | 3589.8 | 1794.3 | 2.001 | Procollagen, type V, alpha 1 | Col5a1 |

**metallopeptidase activity**

GO:0008237

(p=0.001332)

| **Affy Id** | **Unigene id** | **Mean intensity of Solid PCT** | **Mean intensity of TC-PCT** | **Fold Difference** | **Description** | **Gene Symbol** |
| --- | --- | --- | --- | --- | --- | --- |
| 100484_at | Mm.5022 | 706.8 | 50.4 | 14.024 | Matrix metalloproteinase 13 | Mmp13 |
| 100751_at | Mm.3037 | 679.9 | 2070.3 | 0.328 | A disintegrin and metalloprotease domain 10 | Adam10 |
| 103024_at | Mm.15969 | 843.9 | 49.9 | 16.912 | A disintegrin and metalloprotease domain 8 | Adam8 |
| 103554_at | Mm.89940 | 578.2 | 51.3 | 11.271 | A disintegrin and metalloproteinase domain 19 (meltrin beta) | Adam19 |
| 160118_at |  | 3844.3 | 592.5 | 6.488 | matrix metalloproteinase 14 (membrane-inserted) | Mmp14 |
| 161224_f_at | Mm.754 | 64.9 | 20.9 | 3.105 | Angiotensin converting enzyme | Ace |
| 92414_at | Mm.323601 | 112.9 | 37 | 3.051 | RIKEN cDNA 6030403N03 gene | Adam12 |
| 95339_r_at | Mm.2055 | 795.5 | 129 | 6.167 | Matrix metalloproteinase 12 | Mmp12 |
| 95557_at | Mm.27757 | 1466.7 | 232.3 | 6.314 | Bone morphogenetic protein 1 | Bmp1 |
| 98833_at | Mm.4993 | 735 | 44.2 | 16.629 | Matrix metalloproteinase 3 | Mmp3 |
| 99643_f_at | Mm.31395 | 653.2 | 10.8 | 60.481 | Carboxypeptidase E | Cpe |
| 99993_at | Mm.4487 | 900 | 128.3 | 7.015 | Alanyl (membrane) aminopeptidase | Anpep |
| 99474_at | Mm.143889 | 3073.5 | 1747.7 | 1.759 | A disintegrin and metalloprotease domain 5 | Adam5 |

**insulin-like growth factor binding**

GO:0005520

(p=0.000152)

| **Affy Id** | **Unigene Id** | **Mean intensity of Solid PCT** | **Mean intensity of TC-PCT** | **Fold difference** | **Description** | **Gene Symbol** |
| --- | --- | --- | --- | --- | --- | --- |
| 94222_at | Mm.233799 | 838.9 | 163.5 | 5.131 | Insulin-like growth factor binding protein 4 | Igfbp4 |
| 100507_at | Mm.5167 | 175.9 | 46.3 | 3.799 | Nephroblastoma overexpressed gene | Nov |
| 93294_at | Mm.1810 | 1606.1 | 16.3 | 98.534 | Connective tissue growth factor | Ctgf |
| 100566_at | Mm.309617 | 542.1 | 104.3 | 5.198 | Insulin-like growth factor binding protein 5 | Igfbp5 |
| 95083_at | Mm.29254 | 8179.6 | 26.8 | 305.209 | Insulin-like growth factor binding protein 3 | Igfbp3 |
| 92777_at | Mm.1231 | 302.4 | 63.7 | 4.747 | Cysteine rich protein 61 | Cyr61 |

**complement activation, classical pathway**

GO:0006958

(p=0.000461)

| **Affy Id** | **Unigene Id** | **Mean intensity of Solid PCT** | **Mean Intensity of TC-PCT** | **Fold difference** | **Description** | **Gene Symbol** |
| --- | --- | --- | --- | --- | --- | --- |
| 96020_at | Mm.2570 | 5659.2 | 52.9 | 106.979 | Complement component 1, q subcomponent, beta polypeptide | C1qb |
| 162366_r_at | Mm.283217 | 894.9 | 314 | 2.85 | Complement component 2 (within H-2S) | C2 |
| 98562_at | Mm.370 | 7754.5 | 96.6 | 80.274 | Complement component 1, q subcomponent, alpha polypeptide | C1qa |
| 99081_at | Mm.38888 | 5735.8 | 383.5 | 14.956 | Serine (or cysteine) proteinase inhibitor, clade G, member 1 | Serping1 |
| 92223_at | Mm.3453 | 9192.6 | 172.9 | 53.167 | Complement component 1, q subcomponent, gamma polypeptide | C1qg |
| 93497_at | Mm.19131 | 2370.6 | 306 | 7.747 | Complement component 3 | C3 |

**protein kinase activity**

GO:0004672

(p=0.001415)

| **Affy Id** | **Unigene Id** | **Mean intensities of Solid PCT** | **Mean intensities of TC-PCT** | **Fold difference** | **Description** | **Gene symbol** |
| --- | --- | --- | --- | --- | --- | --- |
| 100155_at | Mm.5021 | 662.7 | 204.8 | 3.236 | discoidin domain receptor family, member 1 | Ddr1 |
| 100450_r_at | Mm.279542 | 1186.9 | 685.9 | 1.73 | activin A receptor, type II-like 1 | Acvrl1 |
| 101148_at | Mm.291554 | 219.6 | 515.5 | 0.426 | protein kinase C, iota | Prkci |
| 101842_g_at | Mm.8534 | 214.1 | 84.1 | 2.546 | epidermal growth factor receptor | Egfr |
| 104265_at | Mm.285 | 505.1 | 149.2 | 3.385 | kinase insert domain protein receptor | Kdr |
| 104417_at | Mm.3291 | 681.3 | 155.8 | 4.373 | FMS-like tyrosine kinase 4 | Flt4 |
| 160867_at | Mm.4146 | 295 | 61.1 | 4.828 | platelet derived growth factor receptor, beta polypeptide | Pdgfrb |
| 161067_at | Mm.276018 | 222.8 | 888.2 | 0.251 | tribbles homolog 3 (Drosophila) | Trib3 |
| 92698_at | Mm.239655 | 297.1 | 142.2 | 2.089 | c-mer proto-oncogene tyrosine kinase | Mertk |
| 93431_at | Mm.6529 | 968.7 | 560 | 1.73 | dystrophia myotonica-protein kinase | Dmpk |
| 93483_at | Mm.715 | 895 | 144 | 6.215 | hemopoietic cell kinase | Hck |
| 95079_at | Mm.221403 | 1645.1 | 96.5 | 17.048 | platelet derived growth factor receptor, alpha polypeptide | Pdgfra |
| 97096_at | Mm.253102 | 543.9 | 1072.6 | 0.507 | protein kinase, cAMP dependent regulatory, type II alpha | Prkar2a |
| 97363_at | Mm.254494 | 541.8 | 76.2 | 7.11 | PTK2 protein tyrosine kinase 2 | Ptk2 |
| 97509_f_at | Mm.265716 | 384 | 60.9 | 6.305 | fibroblast growth factor receptor 1 | Fgfr1 |
| 97890_at | Mm.28405 | 725.8 | 88.9 | 8.164 | serum/glucocorticoid regulated kinase | Sgk |
| 98453_at | Mm.3464 | 454.3 | 88.2 | 5.151 | FMS-like tyrosine kinase 1 | Flt1 |
| 98911_at | Mm.289657 | 1199.9 | 155.4 | 7.721 | Janus kinase 1 | Jak1 |
| 99384_at |  | 1514.4 | 4818.6 | 0.314 | proviral integration site 1 | Pim1 |
| 99916_at | Mm.341677 | 86.7 | 19.7 | 4.401 | protein kinase C, eta | Prkch |
| 99936_at | Mm.4345 | 451.4 | 37.9 | 11.91 | tyrosine kinase receptor 1 | Tie1 |
| 99956_at | Mm.247073 | 392.2 | 83.1 | 4.72 | kit oncogene | Kit |
| 99960_at | Mm.27491 | 665.4 | 1131.2 | 0.588 | mitogen activated protein kinase kinase 4 | Map2k4 |
| 160746_at | Mm.6710 | 876.1 | 1579.9 | 0.555 | Rho-associated coiled-coil forming kinase 1 | Rock1 |
| 161233_at | Mm.347478 | 977 | 2629 | 0.372 | proviral integration site 2 | Pim2 |
| 161832_r_at | Mm.22574 | 1699.3 | 247 | 6.88 | colony stimulating factor 1 receptor | Csf1r |
| 93210_g_at | Mm.251494 | 32.7 | 101.1 | 0.323 | NIMA (never in mitosis gene a)-related expressed kinase 4 | Nek4 |
| 94003_at | Mm.333349 | 263.9 | 703.5 | 0.375 | WNK lysine deficient protein kinase 1 | Wnk1 |
| 95721_at | Mm.221235 | 516.3 | 1312.1 | 0.393 | MAP kinase-activated protein kinase 2 | Mapkapk2 |
| 97095_at | Mm.2185 | 121.3 | 610 | 0.199 | Budding uninhibited by benzimidazoles 1 homolog (S. cerevisiae), mRNA (cDNA clone MGC:18385 IMAGE:3671932) | Bub1 |
| 99511_at | Mm.207496 | 591.9 | 286.3 | 2.067 | protein kinase C, beta 1 | Prkcb1 |

**Cell adhesion**

GO:0007155

(p<1 e-06)

| **Affy Id** | **Unigene Id** | **Mean intensity of Solid PCT** | **Mean intensity of TC-PCT** | **Fold difference** | **Description** | **Gene Symbol** |
| --- | --- | --- | --- | --- | --- | --- |
| 92414_at | Mm.323601 | 112.9 | 37 | 3.051 | a disintegrin and metallopeptidase domain 12 (meltrin alpha) | Adam12 |
| 100412_g_at | Mm.4665 | 1037.4 | 54.1 | 19.176 | AE binding protein 1 | Aebp1 |
| 100155_at | Mm.5021 | 662.7 | 204.8 | 3.236 | discoidin domain receptor family, member 1 | Ddr1 |
| 97773_at | Mm.29798 | 418.5 | 68.4 | 6.118 | CD34 antigen | Cd34 |
| 95661_at | Mm.210676 | 1770.1 | 256.9 | 6.89 | CD9 antigen | Cd9 |
| 100006_at | Mm.1571 | 127.9 | 43.6 | 2.933 | cadherin 11 | Cdh11 |
| 102852_at | Mm.257437 | 91.5 | 21.2 | 4.316 | cadherin 2 | Cdh2 |
| 102262_r_at | Mm.300931 | 103.7 | 40.8 | 2.542 | procollagen, type XIII, alpha 1 | Col13a1 |
| 99637_at | Mm.233547 | 1625.1 | 119.5 | 13.599 | procollagen, type XV | Col15a1 |
| 99638_at | Mm.4352 | 2718.2 | 301.7 | 9.01 | procollagen, type XVIII, alpha 1 | Col18a1 |
| 98331_at | Mm.249555 | 669 | 17.4 | 38.448 | procollagen, type III, alpha 1 | Col3a1 |
| 101093_at | Mm.738 | 8683.1 | 77.9 | 111.465 | procollagen, type IV, alpha 1 | Col4a1 |
| 101039_at | Mm.181021 | 7148.9 | 247.9 | 28.838 | procollagen, type IV, alpha 2 | Col4a2 |
| 92567_at | Mm.10299 | 6237.8 | 71 | 87.856 | procollagen, type V, alpha 2 | Col5a2 |
| 95493_at | Mm.2509 | 3647.9 | 669.3 | 5.45 | procollagen, type VI, alpha 1 | Col6a1 |
| 101110_at | Mm.7562 | 4906.3 | 92.2 | 53.214 | procollagen, type VI, alpha 3 | Col6a3 |
| 101130_at | Mm.277792 | 9680.2 | 43.8 | 221.009 | procollagen, type I, alpha 2 | Col1a2 |
| 98990_at | Mm.3758 | 800.2 | 329.8 | 2.426 | breast cancer anti-estrogen resistance 1 | Bcar1 |
| 100134_at | Mm.225297 | 5767.4 | 719.6 | 8.015 | endoglin | Eng |
| 100928_at | Mm.249146 | 6155 | 119 | 51.723 | fibulin 2 | Fbln2 |
| 93294_at | Mm.1810 | 1606.1 | 16.3 | 98.534 | connective tissue growth factor | Ctgf |
| 99007_at | Mm.130227 | 1332.5 | 300.6 | 4.433 | flotillin 2 | Flot2 |
| 92852_at | Mm.193099 | 3363.6 | 59.1 | 56.914 | fibronectin 1 | Fn1 |
| 99067_at | Mm.3982 | 764.2 | 122.3 | 6.249 | growth arrest specific 6 | Gas6 |
| 104469_at | Mm.2976 | 380.5 | 47.4 | 8.027 | podoplanin | Pdpn |
| 92777_at | Mm.1231 | 302.4 | 63.7 | 4.747 | cysteine rich protein 61 | Cyr61 |
| 95511_at | Mm.225096 | 161.4 | 48.1 | 3.356 | integrin alpha 6 | Itga6 |
| 161793_at | Mm.258065 | 778.5 | 179.8 | 4.33 | laminin, alpha 4 | Lama4 |
| 93454_at | Mm.681 | 1919.1 | 102.8 | 18.668 | complement component 1, q subcomponent, receptor 1 | C1qr1 |
| 92880_at | Mm.1451 | 8111 | 3340.8 | 2.428 | milk fat globule-EGF factor 8 protein | Mfge8 |
| 100120_at | Mm.4691 | 163.8 | 48.3 | 3.391 | nidogen 1 | Nid1 |
| 93563_s_at | Mm.20348 | 353.5 | 42.1 | 8.397 | nidogen 2 | Nid2 |
| 95016_at | Mm.271745 | 967.6 | 49.6 | 19.508 | neuropilin 1 | Nrp1 |
| 160469_at | Mm.4159 | 1626.6 | 32.8 | 49.591 | thrombospondin 1 | Thbs1 |
| 94930_at | Mm.26688 | 424.3 | 80.7 | 5.258 | thrombospondin 2 | Thbs2 |
| 92560_g_at | Mm.76649 | 514 | 164.9 | 3.117 | vascular cell adhesion molecule 1 | Vcam1 |
| 160583_at | Mm.272632 | 225.4 | 39.4 | 5.721 | extra cellular link domain-containing 1 | Xlkd1 |
| 96886_at | Mm.220821 | 1198.2 | 115.3 | 10.392 | stabilin 1 | Stab1 |
| 96774_at | Mm.210018 | 343.5 | 71.1 | 4.831 | pleckstrin homology domain containing, family C (with FERM domain) member 1 | Plekhc1 |
| 102327_at |  | 550.7 | 181.7 | 3.031 | amine oxidase, copper containing 3 | Aoc3 |
| 93063_at |  | 7851.8 | 235.2 | 33.384 | amyloid beta (A4) precursor protein | App |
| 101113_at | Mm.318359 | 1261.9 | 3961.7 | 0.319 | ras homolog gene family, member A | Rhoa |
| 104083_at |  | 2455 | 25.7 | 95.525 | cadherin 5 | Cdh5 |
| 93472_at | Mm.7281 | 3589.8 | 1794.3 | 2.001 | Procollagen, type V, alpha 1 (Col5a1), mRNA | Col5a1 |
| 100308_at |  | 650.1 | 88.9 | 7.313 | procollagen, type VIII, alpha 1 | Col8a1 |
| 100019_at |  | 493.1 | 87.6 | 5.629 | chondroitin sulfate proteoglycan 2 | Cspg2 |
| 101399_at |  | 990.5 | 211.4 | 4.685 | perlecan (heparan sulfate proteoglycan 2) | Hspg2 |
| 103816_at |  | 2112.7 | 637 | 3.317 | F11 receptor | F11r |
