# Supplementary material for: Heterologous Tissue Culture Expression Signature Predicts Human Breast Cancer Prognosis
Source: PLoS One. 2007 Jan 3;2(1):e145. doi: 10.1371/journal.pone.0000145 (PMC1764035; doi:10.1371/journal.pone.0000145)
Supplement: Table S2 — Summary of the class prediction results for NKI training set. Genes significantly different between the classes at 0.001 significance level were used for class prediction. 4160 genes were selected as a classifier for Good vs. Bad group and 1651 genes were selected as a classifier for BEST prognosis group. For the prediction of WORST prognosis group 4700 genes were selected as a classifier. Leave-one-out cross-validation method was used to compute misclassification rate based on 100 random permutations. (0.05 MB DOC) [file pone.0000145.s010.doc]

**Table S2. Summary of the class prediction results for NKI training data set.**

|  |  | **CCP** | | **LDA** | | **1NN** | | **3NN** | | **NC** | | **SVM** | |
| --- | --- | --- | --- | --- | --- | --- | --- | --- | --- | --- | --- | --- | --- |
| Class predictor 1 | | | | | | | | | | | | | |
| Predicted Subclass | | Good | Bad | Good | Bad | Good | Bad | Good | Bad | Good | Bad | Good | Bad |
| Good (N=86) | | 83 | 3 | 82 | 4 | 81 | 5 | 84 | 2 | 82 | 4 | 75 | 7 |
| Bad (N=61) | | 12 | 49 | 11 | 50 | 11 | 50 | 12 | 49 | 13 | 48 | 5 | 56 |
| Correctly classified (%) | | 90 | | 90 | | 89 | | 90 | | 88 | | 92 | |
| Significance of LOOCV | | p<0.01 | | p<0.01 | | p<0.01 | | p<0.01 | | p<0.01 | | p<0.01 | |
| Class predictor 2 | | | | | | | | | | | | | |
| Predicted Subclass | | BEST | Not BEST | BEST | Not BEST | BEST | Not BEST | BEST | Not BEST | BEST | Not BEST | BEST | Not BEST |
| BEST (N=30) | | 27 | 3 | 26 | 4 | 25 | 5 | 25 | 5 | 28 | 2 | 24 | 6 |
| Not BEST (N=117) | | 27 | 90 | 27 | 90 | 16 | 110 | 10 | 107 | 24 | 93 | 5 | 112 |
| Correctly classified (%) | | 80 | | 79 | | 86 | | 90 | | 82 | | 93 | |
| Significance of LOOCV | | P=0.01 | | p=0.01 | | p<0.01 | | p<0.01 | | p=0.01 | | p<0.01 | |
| Class Predictor 3 | | | | | | | | | | | | | |
| Predicted Subclass | | WORST | Not WORST | WORST | Not WORST | WORST | Not WORST | WORST | Not WORST | WORST | Not WORST | WORST | Not WORST |
| WORST (N=32) | | 28 | 4 | 28 | 4 | 30 | 2 | 28 | 4 | 28 | 4 | 29 | 3 |
| Not WORST (N=115) | | 5 | 110 | 5 | 110 | 4 | 111 | 3 | 112 | 6 | 109 | 2 | 113 |
| Correctly classified (%) | | 94 | | 94 | | 96 | | 95 | | 93 | | 97 | |
| Significance of LOOCV | | p<0.01 | | p<0.01 | | p<0.01 | | p<0.01 | | p<0.01 | | p<0.01 | |
