# Supplementary material for: Heterologous Tissue Culture Expression Signature Predicts Human Breast Cancer Prognosis
Source: PLoS One. 2007 Jan 3;2(1):e145. doi: 10.1371/journal.pone.0000145 (PMC1764035; doi:10.1371/journal.pone.0000145)
Supplement: Table S3 — Cox univariate and multivariate analysis of risk factors for death. Parameters showing significance in the Cox proportional hazard model are shown in bold. Age is categorized into two groups based on >45 years old or not. Lymph node status is categorized into two groups, one with one or more tumor cells infiltrated into the lymph node and another group with no tumor cells infiltrated into the lymph node. P value for statistical significance was calculated by log-rank test. T1T2 is categorized based on the diameter of tumor size are >2 cm or not. (0.05 MB DOC) [file pone.0000145.s011.doc]

**Table S3. Cox univariate and multivariate analysis of risk factors for death.**

Table S3A. Cox univariate analysis of risk factors for death.

|  | Hazard Ratio | 95% Confidence Interval | | P value |
| --- | --- | --- | --- | --- |
|  |  | CI lower | CI upper |  |
| GOOD vs. Rest of all | 0.772 | 0.485 | 1.23 | 0.275 |
| BEST vs. Rest of all | 0.147 | 0.054 | 0.402 | 1.47E-05 |
| WORST vs. Rest of all | 3.16 | 1.98 | 5.05 | 3.79E-07 |
| T1T2 (>2cm vs. <= 2cm) | 2.13 | 1.35 | 3.37 | 0.0009 |
| Age (>45 years vs. <=45 years) | 0.711 | 0.449 | 1.13 | 0.146 |
| Lymph node status (0 vs. >=1 cells ) | 0.877 | 0.563 | 1.366 | 0.56 |
| Tumor grade I vs. grade II+ III | 7.38 | 2.7 | 20.2 | 4.77e-06 |
| Tumor grade III vs. grade I+ II | 0.299 | 0.188 | 0.474 | 5.29e-08 |
| Mastectomy | 1.203 | 0.773 | 1.871 | 0.412 |
| Chemo therapy | 0.790 | 0.494 | 1.264 | 0.32 |
| Hormonal Therapy | 0.606 | 0.262 | 1.40 | 0.235 |
| Estrogen Receptor status | 3.28 | 2.1 | 5.14 | 3.42E-08 |

Table S3B. Multivariate analysis of risk factors for death.

|  | Hazard  ratio | 95% Confidence Interval | | P value |
| --- | --- | --- | --- | --- |
|  |  | CI lower | CI upper |  |
| GOOD vs. Rest of samples | 0.823 | 0.4445 | 1.526 | 0.540 |
| BEST vs. Rest of samples | 0.247 | 0.0779 | 0.783 | **0.017** |
| WORST vs. Rest of samples | 1.111 | 0.5408 | 2.282 | 0.770 |
| T1T2 (>2cm vs. <= 2cm) | 1.695 | 1.0366 | 2.770 | **0.049** |
| Age (>45 years vs. <=45 years) | 0.658 | 0.4108 | 1.053 | 0.081 |
| Lymph node status(0 vs. >=1 cells ) | 1.465 | 0.7301 | 2.940 | 0.280 |
| Tumor grade I vs. grade II+ III | 3.504 | 1.1986 | 10.242 | **0.022** |
| Tumor grade III vs. grade I+ II | 0.873 | 0.4922 | 1.549 | 0.640 |
| Estrogen Receptor status | 1.709 | 0.8862 | 3.296 | 0.110 |
| Chemo therapy | 0.638 | 0.3092 | 1.318 | 0.230 |
| Hormonal therapy | 0.645 | 0.2644 | 1.576 | 0.340 |
| Mastectomy | 1.273 | 0.8098 | 2.000 | 0.290 |

Parameters showing significance in the Cox proportional hazard model are shown in bold. Age is categorized into two groups based on > 45 years old or not. Lymph node status is categorized into two groups, having one or more tumor cells infiltrated into lymph node as one group and another group having no tumor cells infiltrated into lymph node. P value for statistical significance was calculated by log-rank test. T1T2 is categorized based on the diameter of tumor size are >2cm or not.
